# BN‐ and BO‐Doped Inorganic–Organic Hybrid Polymers with Sulfoximine Core Units

**DOI:** 10.1002/chem.201903289

**Published:** 2019-09-09

**Authors:** Felix Brosge, Thomas Lorenz, Holger Helten, Carsten Bolm

**Affiliations:** ^1^ Institute of Organic Chemistry RWTH Aachen University Landoltweg 1 52074 Aachen Germany; ^2^ Institute of Inorganic Chemistry RWTH Aachen University Landoltweg 1 52074 Aachen Germany; ^3^ Institute of Inorganic Chemistry Julius-Maximilians-Universität Würzburg Am Hubland 97074 Würzburg Germany

**Keywords:** boron, hybrid materials, polymers, sulfoximines, sulfur

## Abstract

While polysulfones constitute a class of well‐established, highly valuable applied materials, knowledge about polymers based on the related sulfoximine group is very limited. We have employed functionalized diaryl sulfoximines and a *p*‐phenylene bisborane as building blocks for unprecedented BN‐ and BO‐doped alternating inorganic–organic hybrid copolymers. While the former were accessed by a facile silicon/boron exchange protocol, the synthesis of polymers with main‐chain B–O linkages was achieved by salt elimination.

## Graphical abstract

**Fit us together**: BN‐ and BO‐doped alternating inorganic–organic hybrid copolymers were synthesized using functionalized diaryl sulfoximines and *p*‐phenylene bisborane as monomer units (see scheme).

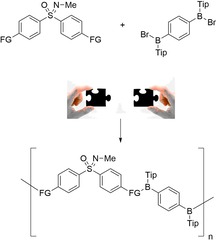

Polysulfones are a family of plastic materials that are noted for their high thermal and oxidative stability.^1^ They are being used within fluid handling components, steam sterilizable biomedical moldings as well as in a range of chemical process and automotive applications.^2^ Some of us recently reported a series of BN‐doped inorganic–organic hybrid polymers,^3^, ^4^, ^5^, ^6^ including the first poly(*p*‐phenylene iminoborane), which can be regarded as a BN‐analogue of poly(*p*‐phenylene vinylene) (PPV).3d A dapson‐type diaryl sulfone was also incorporated into a polymeric material.3e

Formal exchange of a sulfonyl oxygen by a nitrogen atom converts a sulfone into a sulfoximine. The latter compounds are relevant in asymmetric synthesis^7^ and applications in medicinal^8^ and crop protection chemistry.^9^ Functionalizing the sulfoximine nitrogen allows a fine‐tuning of physicochemical properties, which proved useful in drug design and bioactivity adjustment.^10^ Surprisingly, sulfoximines have only once been applied as building blocks in polymers.^11^ In that study, Takata et al. used Friedel–Crafts reactions to prepare polysulfoximines with molecular weights (*M*_n_) of approximately 13 000. Herein, we describe the synthesis and characterization of the first inorganic–organic hybrid polysulfoximines.

In light of previous work,^12^ sulfoximines **1** and **2** were identified as suitable organic starting materials. Both compounds were N‐methylated, thereby confining the reactive anchor sites of the molecules to the free arylic amino and hydroxyl groups. With the vision to allow future variations of the N‐substituent, phthalimid‐ and benzyl‐protected NH‐sulfoximines **4** and **7**, respectively, were targeted first. The synthetic sequences are shown in Scheme 1. The preparation of **1** started from known diarylsulfide **3**,^12^ which was imidated and oxidized by adopting a protocol reported by Luisi, Bull, and others^13^ to give **4** in 62 % yield. Noteworthy, we applied aqueous ammonia as a nitrogen source instead of the originally suggested ammonium carbamate.

**Scheme 1:**
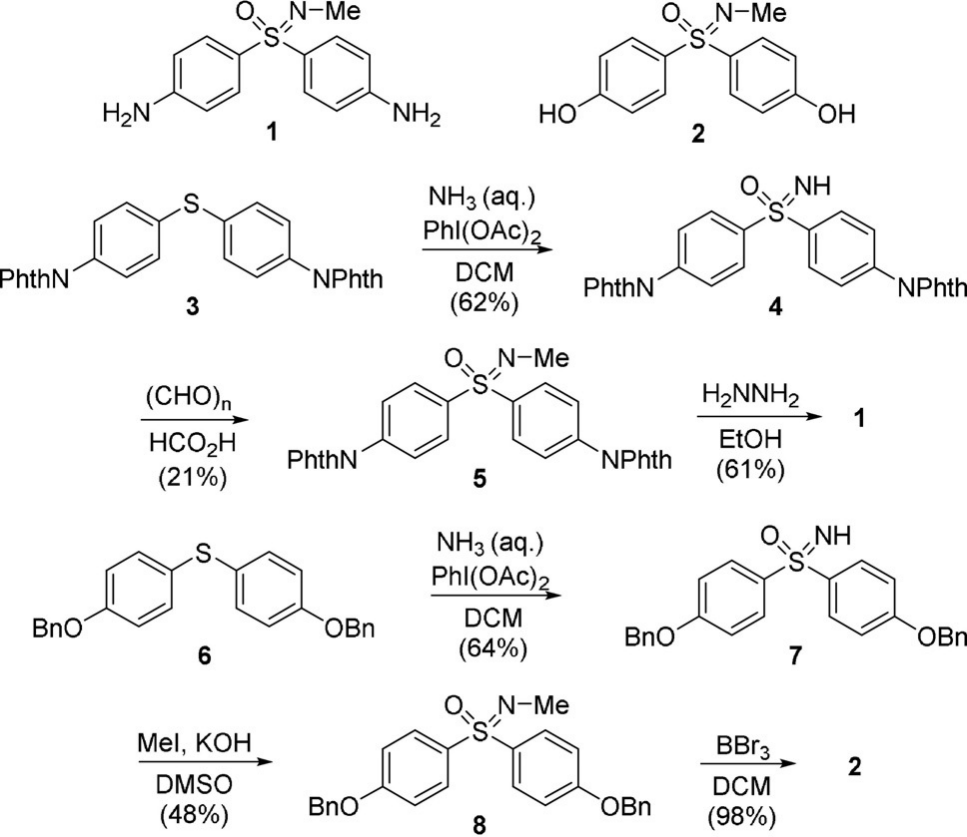
Syntheses of key intermediates **1** and **2**.

N‐Methylation under standard Eschweiler–Clark conditions afforded sulfoximine **5** (21 %), which was deprotected with hydrazine in ethanol to give **1** in 61 % yield. Following an analogous reaction sequence, sulfoximine **2** was prepared by imidation/oxidation of **6** 14 to give **7** (64 %) followed by N‐methylation with MeI in KOH/DMSO^15^ providing **8** in 48 % yield and sequential debenzylation with BBr_3_ (98 %).

Targeting a polymer formation by silicon/boron exchange, bis(silylated) sulfoximine **9** was prepared, in analogy of literature precedence,^16^ by treatment of **1** with a mixture of Me_3_SiCl and Et_3_N in THF at 45 °C for 24 h. The coupling partner for **9** was bis(bromoborane) **10** 3d (Tip=2,4,6‐triisopropylphenyl). Two co‐polycondensation reactions were performed (Scheme 2). In both cases, a 1:1 ratio of **9** and **10** was applied. In the first experiment (trial 1), the mixture was kept in dichloromethane for 3 days at ambient temperature. Trial 2 involved *o*‐difluorobenzene (*o*‐DFB) as the solvent and heating the mixture to 80 °C for 24 h. The resulting alternating copolymers **11** were then purified by precipitation from concentrated solution with hexane and subsequent drying in vacuo. The identities of copolymers **11**, which were obtained as off‐white solids, were unambiguously ascertained by NMR spectroscopy. Their molecular mass distributions were determined by gel permeation chromatography (GPC, Table 1). For both samples, the ^1^H NMR spectrum showed a shift of the N*H*‐Signal from *δ*=3.77 ppm in **9** to the aromatic region in **11** (*δ*=7.25 ppm), which was also observed in previously prepared related BN polymers.3d

**Scheme 2:**
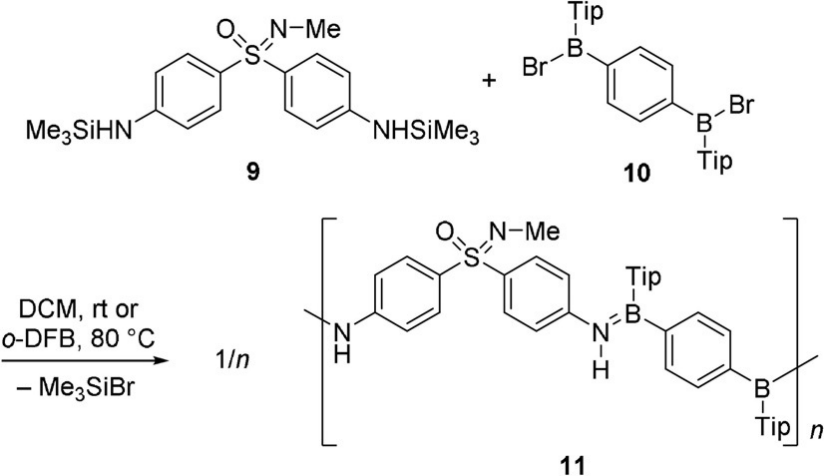
Polycondensation reaction of sulfoximine **9** and bisborane **10** to give alternating copolymer **11**.

**Table 1:** GPC data of polymers **11** and **15** (against polystyrene standards).

	*M*_n_	*M*_W_	PDI	DP_n_
**11** (trial 1)^[a]^	9 750	18 600	1.91	13
**11** (trial 2)^[b]^	11 830	28 900	2.44	15
**15** (trial 1)^[a]^	2400	2970	1.52	3
**15** (trial 2)^[b]^	5300	9740	1.84	7

[a] Prepared in dichloromethane (DCM), rt, 3 d. [b] Prepared in *o*‐difluorobenzene (*o*‐DFB), 80 °C, 24 h.

The GPC analyses revealed number average molecular weights of *M*_n_=9 750 (trial 1) and 11 830 (trial 2), according to polymerization degrees of DP_*n*_=13 and 15, respectively. The polydispersity indices were close to 2, as expected for step‐growth polycondensation processes.

Next, copolymers with main‐chain B−O linkages^17^ were targeted. Hypothesizing that such products could be accessed by analogous Si/B exchange reactions as applied before in the synthesis of **11**, organic starting materials with silylated phenolic hydroxyl groups became of interest. In order to get an estimate of the feasibility of such an approach, a prior model reaction between **10** and trimethylsilylated phenol **12** was performed (Scheme 3). In the first experiment, the reaction was run in dichloromethane at room temperature. As hypothesized, product **14** was indeed formed as revealed by ^1^H and ^11^B NMR spectroscopy. The initial presence of two doublets (*δ*=8.04 and 7.88 ppm) in the ^1^H NMR spectrum suggested a stepwise formation of **14**. However, the entire process was very slow, and even after four weeks the conversion was not yet complete. A similar outcome resulted when *o*‐difluorobenzene was used as a solvent at a reaction temperature of 80 °C. Also in this case, the conversion was slow, taking five weeks in total. Although these results showed that a Si/B exchange could, in principle, be applied to accomplish a B−O bond formation starting from **10**, the slow rate of this process proved unfavorable for its application to co‐polycondensation reactions. Therefore, we decided to investigate B−O bond formation between **10** and the parent free phenol (**13**).

**Scheme 3:**
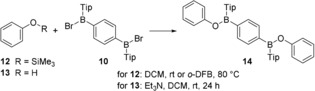
Model reactions testing the feasibility of Si/B exchange and salt elimination reactions in the formation of alternating copolymers with B−O linkages.

While initial attempts without base remained unsatisfying, the presence of triethylamine had a tremendously positive effect, leading to a clean and quantitative coupling providing **14** within 24 hours at room temperature. Compound **14** was then isolated by filtration and characterized by multinuclear NMR spectroscopy. The ^11^B{^1^H} NMR spectrum showed a resonance at *δ*=47.3 ppm, which is in the expected range for the suggested constitution.

Encouraged by this result, the aforementioned conditions were applied in the copolymerization of sulfoximine **2** with bisborane **10** (Scheme 4). Within three days in the presence of Et_3_N, the dichloromethane solution became highly viscous (trial 1). However, after work‐up the GPC analysis revealed that the product was of relatively low molecular weight (*M*_n_=2 400, DP_*n*_=3; Table 1). Consequently, in the next experiment (trial 2) the solvent was changed to *o*‐DFB, and then the reaction temperature was raised to 80 °C. Pleasingly, in this manner, after 24 h the molecular weight (*M*_n_) of the resulting polymer **15** was increased to 5 300, revealing an average chain length of DP_*n*_=7 (Table 1).^18^

**Scheme 4:**
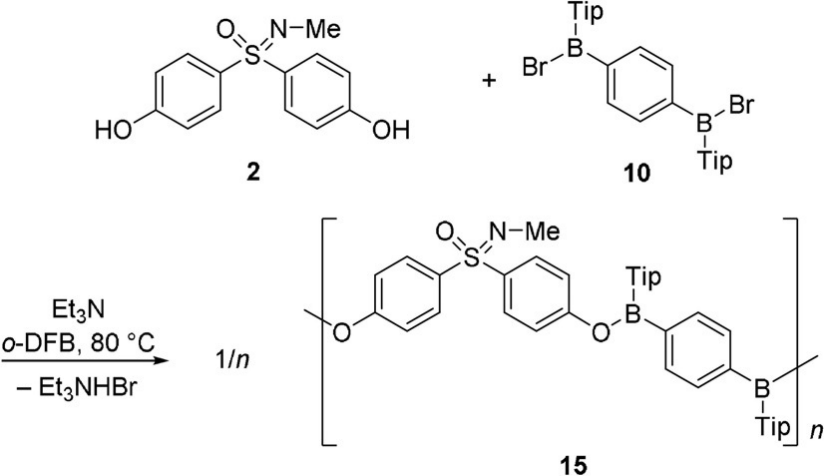
Polycondensation reaction of sulfoximine **2** and bisborane **10** to give alternating copolymer **15**.

In summary, we have prepared the first inorganic–organic hybrid sulfoximine‐containing polymers as alternating copolymers with B=N and B−O linkages. While our Si/B exchange polycondensation protocol was successful in the former case, for the synthesis of polymers with B−O linkages in the main chain a salt elimination approach proved to be favorable. In view of the recently demonstrated advantageous effect of the formulation of dapsone‐type drugs into polymer conjugates for anti‐inflammatory purposes^19^ on the one hand, and the well‐established biomedical activity of many boron‐containing polymers^20^ on the other hand, we are currently exploring the biomedical potential of our novel sulfoximine‐B=N/B−O hybrids in detail.

## Conflict of interest

The authors declare no conflict of interest.

## Supporting information

As a service to our authors and readers, this journal provides supporting information supplied by the authors. Such materials are peer reviewed and may be re‐organized for online delivery, but are not copy‐edited or typeset. Technical support issues arising from supporting information (other than missing files) should be addressed to the authors.

Supplementary
