# Supplementary material for: BN‐ and BO‐Doped Inorganic–Organic Hybrid Polymers with Sulfoximine Core Units
Source: Chemistry. 2019 Sep 9;25(55):12708–11. doi: 10.1002/chem.201903289 (PMC6790941; doi:10.1002/chem.201903289)
Supplement: Supplementary file 1 — Supplementary [file CHEM-25-12708-s001.pdf]

CHEMISTRY
A European Journal

Supporting Information

BN- and BO-Doped Inorganic–Organic Hybrid Polymers with Sulfoximine Core Units

Felix Brosge+,[a] Thomas Lorenz+,[b] Holger Helten,*[b] and Carsten Bolm*[a]

chem_201903289_sm_miscellaneous_information.pdf

Electronic Supplementary Information for:

BN-and BO-Doped Inorganic-Organic Hybrid Polymers with Sulfoximine Core Units

Felix Brosge,§[a] Thomas Lorenz,§[b] Holger Helten,*[b] and Carsten Bolm*[a]

[a] Institute of Organic Chemistry, RWTH Aachen University, Landoltweg 1, 52074 Aachen, Germany

[b] Institute of Inorganic Chemistry, RWTH Aachen University, Landoltweg 1, 52074 Aachen, Germany,

New address: Institute of Inorganic Chemistry, Julius-Maximilians-Universität-Würzburg, Am Hubland, 97074 Würzburg, Germany

§These authors contributed equally.

Table of Content

1. General information S2

2. Syntheses and characterisations of the *N*-methylated diphenyl sulfoximine monomers S3

3. References S7

4. NMR spectra S8

5. GPC traces S21

1. General information

Unless otherwise stated, all reagents were purchased from commercial suppliers and used without further purification.
All solvents were distilled prior to using. When required, solvents were dried according to general purification methods. Reactions were tracked by thin layer chromatography using aluminum foil backed silica TLC plates with a fluorescent indicator from Merck. UV-active compounds were detected with a UV lamp (λ = 254 nm). For flash column chromatography (FCC), silica gel 60 (63–200 μm) was used as stationary phase. ^{1}H and ^{13}C NMR spectra were recorded either on a Varian V-NMRS 600, Varian V-NMRS 400 or Varian Mercury 300 in deuterated solvents. The chemical shifts (δ) are given in ppm relative to the residual peak of the non-deuterated solvent as internal standard (^{1}H: $CDCl_3$, 7.26 ppm; DMSO-d_6, 2.50 ppm; MeCN-d_3, 1.94 ppm; acetone-d_6, 2.05 ppm; ^{13}C: $CDCl_3$, 77.16 ppm; DMSO-d_6, 39.52 ppm; MeCN-d_3, 118.26 ppm; acetone-d_6, 29.84 ppm) or external $BF_3 \cdot OEt_2$ (^{11}B). ^{13}C{^{1}H} NMR spectra were recorded at 100 or 151 MHz with complete proton decoupling and will be stated as ^{13}C for simplification.
Spin-spin coupling constants (J) are given in Hz. Coupling patterns are given as br s (broad singlet), s (singlet), t (triplet), q (quartet), sept (septet) and m (multiplet). Data are reported as follows: Chemical shift, multiplicity and integration.
The IR spectra were recorded on a PerkinElmer Spectrum 100 spectrometer with an attached UATR device Diamond KRS-5. All IR data were collected by attenuated total reflectance (ATR) and wave numbers ν are given in cm^{-1}. Mass spectra were recorded on a Finnigan SSQ 7000 spectrometer (EI, 70 eV). High resolution mass spectra (HRMS) were recorded on a Thermo Scientific LTQ Orbitrap XL spectrometer.
Melting points (mp) were measured on a Büchi B-560 melting point apparatus.

2. Syntheses and characterisations of the *N*-methylated diphenyl sulfoximine monomers

2,2′-[4,4′-Thiobis(1,4-phenylene)]diisoindoline-1,3-dione (3)

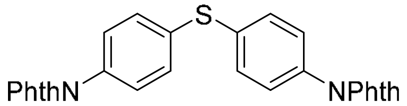

To a solution of 4,4'-thiodianiline (6.00 g, 27.7 mmol) in AcOH (111 mL) phthalic anhydride (9.04 g, 61.0 mmol) was added. The reaction mixture was heated to reflux overnight. After cooling to room temperature the solvent was removed under reduced pressure. The title compound **3** (12.4 g, 25.8 mmol, 93%) was isolated after purification by FCC (1–2% EtOAc in DCM) as a white solid.

^{1}H NMR (600 MHz, DMSO-*d*$_6$) δ = 8.00–7.97 (m, 4H), 7.93–7.90 (m, 4H), 7.55–7.50 (m, 8H) ppm.

^{13}C{^{1}H} NMR (151 MHz, DMSO-*d*$_6$) δ = 166.9, 134.8, 134.3, 131.5, 131.3, 131.2, 128.4, 123.5 ppm.

The NMR data is in accordance with the literature.[1]

Bis[4-(benzyloxy)phenyl]sulfide (6)

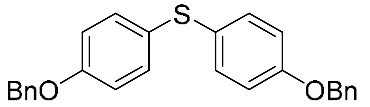

To an ice cold solution of 4,4'-thiodiphenol (5.00 g, 22.9 mmol) in dry THF (90 mL) was added NaH (60% in petrol oil, 2.29 g, 57.3 mmol). The reaction mixture was stirred for 30 min at 0 °C. Then benzyl bromide (10.9 mL, 91.7 mmol) was added. The reaction mixture was stirred overnight at room temperature. Ice was added and the aqueous phase was extracted three times with DCM. The combined organic phases were washed with water, dried over $MgSO_4$ and filtered. The solvent was removed under reduced pressure. The title compound **6** (8.46 g, 21.2 mmol, 93%) was isolated after purification by FCC (10–50% DCM in pentane) as a white solid.

^{1}H NMR (400 MHz, $CDCl_3$) δ= 7.43–7.27 (m, 14H), 6.93–6.90 (m, 4H), 5.04 (s, 4H) ppm.

^{13}C{^{1}H} NMR (101 MHz, $CDCl_3$) δ= 158.3, 136.9, 132.9, 128.8, 128.2, 127.8, 127.6, 115.8, 70.3 ppm.

The NMR data is in accordance with the literature.[2]

General procedure for the synthesis of *N*H sulfoximines from sulfides (GP 1):

The sulfide (**3** or **6**, 1.0 equiv.) was dissolved in DCM (5 mL/mmol). An aqueous solution of NH_3 (13.5 M, 4.0 equiv.) and (diacetoxyiodo)benzene (2.5 equiv.) were added at room temperature, and the reaction mixture was stirred overnight. The solvent was either removed under reduced pressure (**4**, variant A) or water was added to the reaction mixture and it was extracted three times with DCM. The combined organic layers were washed with water and dried over $MgSO_4$, filtered and then the solvent was removed under reduced pressure (**7**, variant B). The corresponding product (**4** or **7**) was isolated after purification by FCC (5–15% EtOAc in DCM).

2,2'-[Sulfonimidoylbis(1,4-phenylene)]bis(isoindoline-1,3-dione) (4)

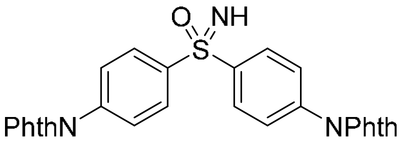

Prepared according to GP 1 (variant A) from 2,2′-[4,4′-thiobis(1,4-phenylene)]diisoindoline-1,3-dione (**3**) (5.50 g, 11.5 mmol), (diacetoxyiodo)benzene (9.26 g, 28.8 mmol) and aqueous NH_3 solution (3.41 mL, 46.0 mmol). The title compound **4** (3.61 g, 7.11 mmol, 62%) was isolated after purification by FCC as a white solid; mp: 274–275 °C.

^{1}H NMR (600 MHz, DMSO-*d*$_6$) δ = 8.16–8.15 (m, 4H), 8.00–7.97 (m, 4H), 7.93–7.92 (m, 4H), 7.69–7.68 (m, 4H) ppm.

^{13}C{^{1}H} NMR (151 MHz, DMSO-*d*$_6$) δ = 166.5, 142.7, 135.6, 134.9, 131.5, 128.5, 127.6, 123.6 ppm.

IR (ATR): ν = 3477, 3265, 3067, 2925, 2852, 2680, 2323, 2106, 1996, 1922, 1708, 1590, 1493, 1373, 1222, 1083, 1013, 948, 885, 836, 796, 716, 669.

MS (EI): *m/z* = 145 (14), 143 (39), 108 (23), 91 (100), 77 (21), 65 (13).

MS (CI): *m/z* = 192 (10), 178 (11), 123 (78), 114 (11), 101 (16), 89 (75), 87 (63), 85 (100), 83 (94), 75 (18), 73 (31), 71 (12), 61 (79).

HRMS (ESI): *m/z* calcd. for $C_{28}H_{18}O_5N_3S$: 508.0962; found: 508.0953.

Bis(4-(benzyloxy)phenyl)(imino)-λ^6-sulfanone (7)

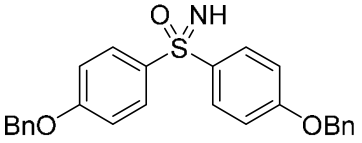

Prepared according to GP 1 (variant B) from bis[4-(benzyloxy)phenyl]sulfide (**6**) (7.50 g, 18.8 mmol), (diacetoxyiodo)benzene (15.1 g, 47.0 mmol) and aqueous NH_3 solution (5.57 mL, 75.2 mmol). The title compound **7** (5.18 g, 12.1 mmol, 64%) was isolated after purification by FCC as a white solid; mp: 159–161 °C.

^{1}H NMR (600 MHz, $CDCl_3$) δ= 7.95–7.94 (m, 4H), 7.39–7.32 (m, 10H), 7.01–7.00 (m, 4H), 5.08 (s, 4H) ppm.

^{113}C{^{1}H} NMR (151 MHz, $CDCl_3$) δ= 162.0, 135.9, 135.3, 129.8, 128.7, 128.3, 127.4, 115.1, 70.3 ppm.

IR (ATR): ν = 3459, 3319, 3025, 2937, 2294, 2101, 1985, 1909, 1739, 1583, 1489, 1376, 1303, 1224, 1093, 977, 828, 747, 699.

MS (EI): *m/z* = 429 ([M]$^+$, 10), 198 (17), 91 (100).

MS (CI): *m/z* = 458 (22), 431 (26), 430 ([M+H]$^+$, 91), 248 (40), 246 (11), 185 (13), 107 (28), 104 (12), 93 (18), 92 (24), 91 (100).

HRMS (ESI): *m/z* calcd. for $C_{26}H_{23}O_3NNaS$: 452.1291; found: 452.1282.

2,2'-[(Methylsulfonimidoyl)bis(1,4-phenylene)]bis(isoindoline-1,3-dione) (**5**)

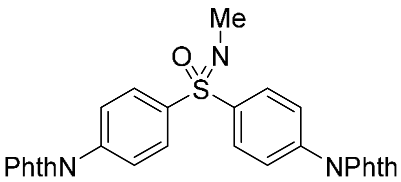

To a suspension of 2,2'-[sulfonimidoylbis(1,4-phenylene)]bis(isoindoline-1,3-dione) (**4**) (200 mg, 0.394 mmol) in formic acid (0.5 mL) was added *p*-Formaldehyde (17.6 mg, 0.586 mmol). The reaction mixture was stirred at 120 °C for 36 h. After the reaction was cooled to room temperature the solvent was removed under reduced pressure. The title compound **5** (43.6 mg, 0.0836 mmol, 21%) was isolated after purification by FCC (2–10% EtOAc in DCM) as a white solid; mp: 234–236 °C.

^{1}H NMR (600 MHz, $CDCl_3$) δ = 8.16–8.14 (m, 4H), 7.99–7.96 (m, 4H), 7.84–7.81 (m, 4H), 7.73–7.72 (m, 4H), 2.91 (s, 3H) ppm.
$^{13}C\{^1H\}$ NMR (151 MHz, $CDCl_3$) δ = 166.7, 136.2, 135.0, 131.5, 129.8, 126.7, 124.2, 29.6 ppm.
IR (ATR): ν = 3481, 3065, 3025, 2923, 2875, 2807, 2325, 2100, 2010, 1930, 1787, 1708, 1590, 1494, 1466, 1373, 1295, 1235, 1155, 1113, 1077, 1016, 955, 880, 834, 798, 749, 716, 674.
MS (EI): *m/z* = 137 (11), 85 (25), 83 (41), 57 (13), 50 (21), 49 (17), 48 (100), 47 (96).
MS (CI): *m/z* = 522 ($[M+H]^+$, 1), 149 (100), 61 (92).
HRMS (ESI): *m/z* calcd. for $C_{29}H_{20}O_5N_3S$: 522.1118; found: 522.1112.

Bis[4-(benzyloxy)phenyl](methylimino)-λ^6-sulfanone (8)

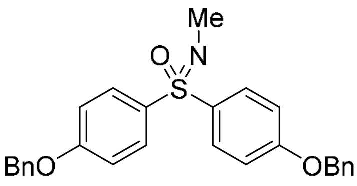

To a solution of bis[4-(benzyloxy)phenyl](imino)-λ^6-sulfanone (**7**) (4.00 g, 9.31 mmol) in dry DMSO (14 mL) was added KOH (1.04 g, 18.6 mmol). The reaction mixture was stirred for 30 min at room temperature. Afterwards MeI (0.868 mL, 14.0 mmol) was added and the reaction mixture was stirred overnight. Upon completion of the reaction, water was added and the aqueous phase was extracted three times with DCM. The combined organic layers were washed with water, dried over $MgSO_4$ and filtered. The solvent was removed under reduced pressure. The title compound **8** (1.98 g, 4.45 mmol, 48%) was isolated after purificaion by FCC (1–5% EtOAc in DCM) as a white solid; mp: 109–111 °C.
^{1}H NMR (600 MHz, $CDCl_3$) δ = 7.91–7.89 (m, 4H), 7.39–7.33 (m, 10H), 7.07–7.04 (m, 4H), 5.09 (s, 4H), 2.82 (s, 3H) ppm.
$^{13}C\{^1H\}$ NMR (151 MHz, $CDCl_3$) δ = 162.6, 135.9, 130.7, 128.9, 128.5, 127.6, 115.7, 115.7, 70.5, 29.2 ppm.
IR (ATR): ν = 3066, 3035, 2922, 2875, 2804, 2322, 2105, 1907, 1736, 1654, 1584, 1491, 1461, 1386, 1301, 1235, 1174, 1140, 1098, 995, 919, 865, 835, 750, 697.
MS (EI): *m/z* = 91 (100).
MS (CI): *m/z* = 444 ($[M+H]^+$, 14), 252 (15), 225 (16), 224 (100).
HRMS (ESI): *m/z* calcd. for $C_{27}H_{26}O_3NS$: 444.1628; found: 444.1625.

Bis(4-aminophenyl)(methylimino)-λ^6-sulfanone (1)

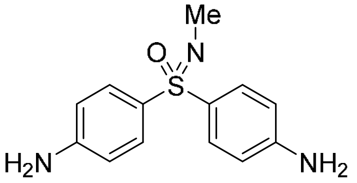

To a solution of 2,2'-[(methylsulfonimidoyl)bis(1,4-phenylene)]bis(isoindoline-1,3-dione) (**5**) (346 mg, 0.663 mmol) in EtOH (7 mL) was added an aqueous hydrazine monohydrate solution (60%, 3.30 mL, 0.66 mmol). The reaction mixture was stirred overnight. Upon completion of the reaction, water was added and the aqueous phase was extracted five times with EtOAc. The combined organic phases were dried over $MgSO_4$, filtered and the solvent was removed under reduced pressure. The title compound **1** (106 mg, 0.404 mmol, 61%) was isolated after purification by FCC (1–5% MeOH in DCM + 0.5% NEt_3) as a white solid; mp: 175–176 °C.
^{1}H NMR (600 MHz, MeCN-d_3) δ = 7.54–7.52 (m, 4H), 6.65–6.63 (m, 4H), 4.69 (s, 4H), 2.61 (s, 3H) ppm.

$^{13}C\{^1H\}$ NMR (151 MHz, MeCN-d_3) δ=152.6, 130.7, 129.3, 114.5, 29.7 ppm.
IR (ATR): ν = 3558, 3478, 3427, 3361, 3312, 3184, 3063, 2967, 2929, 2875, 2805, 2666, 2322, 2169, 2114, 1990, 1911, 1742, 1625, 1590, 1497, 1462, 1400, 1337, 1307, 1258, 1206, 1144, 1070, 993, 940, 912, 861, 809, 753, 716, 696, 660.
MS (EI): *m/z* = 261 ($[M]^+$, 4), 122 (10), 121 (100), 94 (16), 93 (24), 92 (16), 65 (36).
MS (CI): *m/z* = 290 (15), 263 (11), 262 ($[M+H]^+$, 100), 140 (26), 121 (57).
HRMS (ESI): *m/z* calcd. for $C_{13}H_{16}ON_3S$: 262.1009; found: 262.1008.

Bis(4-hydroxyphenyl)(methylimino)-λ⁶-sulfanone (2)

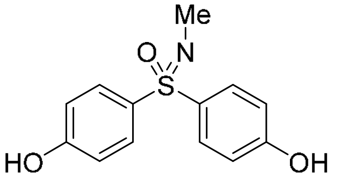

To an ice cold solution of bis[4-(benzyloxy)phenyl]-(methylimino)-λ^6-sulfanone (**8**) (1.33 g, 5.19 mmol) in dry DCM (60 mL) was added BBr_3 (1 M in DCM, 10.4 mL, 10.4 mmol). The reaction mixture was stirred at room temperature for 30 min. Upon completion of the reaction, ice was added and the solvent was removed under reduced pressure. The title compound **2** (1.33 g, 5.07 mmol, 98%) was isolated after purification by FCC (3–10% MeOH in DCM + 0.1% NEt_3) as a white solid; mp: 60 °C.
^1H NMR (600 MHz, Acetone-d_6) δ = 9.23 (br s, 2H), 7.78–7.76 (m, 4H), 6.95–6.93 (m, 4H), 2.66 (s, 3H) ppm.
$^{13}C\{^1H\}$ NMR (151 MHz, Acetone-d_6) δ = 161.7, 132.9, 131.4, 116.5, 29.5 ppm.
IR (ATR): ν = 3275, 2924, 2803, 2671, 2577, 2473, 2178, 2103, 1985, 1911, 1699, 1577, 1492, 1443, 1376, 1283, 1222, 1141, 1087, 831, 718, 691.
MS (EI): *m/z* = 263 ($[M]^+$, 6), 182 (27), 142 (15), 141 (18), 123 (10), 122 (100), 93 (31), 82 (46), 81 (13), 80 (47), 79 (17), 65 (42), 63 (15), 55 (11), 53 (12).
MS (CI): *m/z* = 242 (16), 241 (100), 214 (11), 213 (60), 182 (34), 156 (23), 141 (55), 125 (13), 111 (11), 79 (13).
HRMS (ESI): *m/z* calcd. for $C_{13}H_{14}O_3NS$: 264.0689; found: 264.0679.

(Methylimino)bis(4-((trimethylsilyl)amino)phenyl)- λ⁶-sulfanone (9)

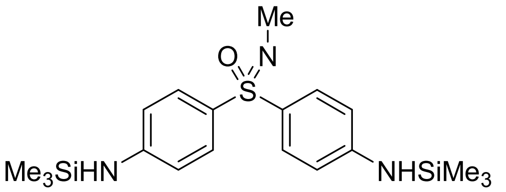

To a solution of **1** (21.4 mg, 0.08 mmol) in DCM (0.7 mL) was added Et_3N (0.2 mL) and TMSCl (0.04 mL, 0.32 mmol). The reaction mixture was heated at 45 °C for 24 h. The solvent was removed in vacuo. The product was extracted with hexane (3 x 3 mL) and the solvent was removed in vacuo. **11** was isolated as white solid (27.6 mg, 0.068 mmol, 85%).
^1H-NMR (400 MHz, $CDCl_3$) δ = 7.68–7.66 (m, 4H), 6.64–6.2 (m, 4H), 3.77 (s, 2H, N*H*), 2.78 (s, 3H, NCH_3), 0.27 (s, 18H, Si(CH_3)$_3$) ppm.

Synthesis of polymer 11 (trial 2)

To a suspension of **10** (40.5 mg, 0.10 mmol) in *o*-DFB (0.5 mL) was added a solution of **10** (66.5 mg, 0.10 mmol) in *o*-DFB (0.5 mL). The mixture was heated to 80 °C for 24 h. Then the product was precipitated with hexane. All volatiles were removed, and the product was dried in vacuo.

^{1}H-NMR (400 MHz, $CDCl_3$) δ = 7.61 (s, 4H), 7.59 (s, 4H), 7.25 (br s, 2H, N*H*), 6.97 (s, 4H), 6.91 (d, 4H), 2.92 (sept, 2H), 2.68 (s, 3H, NC*H₃*), 2.53 (sept, 4H), 1.30 (d, 12H), 0.92 (d, 12H), 0.87 (m, 12H) ppm.

Synthesis of 1,4-Bis(phenoxy(2,4,6-triisopropylphenyl)boranyl)benzene (14)

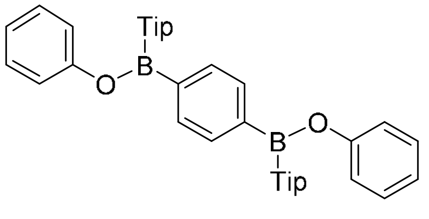

To a solution of **10** (166 mg, 0.25 mmol) in DCM (1.5 mL) was added Et_3N (0.1 mL) and a solution of trimethyl(phenoxy)silane (83 mg, 0.50 mmol) in DCM (0.5 mL). The mixture was stirred for 24 h. Then the solvent was removed in vacuo. The product was extracted with hexane (3 x 2 mL) and the solvent was removed in vacuo. **14** was isolated as a white solid (126 mg, 0.18 mmol, 73 %).

^{1}H-NMR (400 MHz, $CDCl_3$) δ = 7.82 (s, 4H, B-C_6H_4), 7.21–7.17 (m, 4H, Ph), 7.05–6.94 (m, 6H, Ph), 6.89 (s, 4H, C_6H_2), 2.92–2.79 (sept, 2H, C*H*-*i*Pr), 2.66–2.55 (sept, 4H, C*H*-*i*Pr), 1.24 (d, 12H, C*H*₃-*i*Pr), 1.01 (d, 24H, C*H*₃-*i*Pr) ppm.
^{11}B{^{1}H} NMR (128 MHz, $CDCl_3$): δ = 47.3 ppm (s).

Synthesis of polymer 15 (trial 2)

To a suspension of **10**[3] (65 mg, 0.25 mmol) in *o*-DFB (1.5 mL), was added Et_3N (0.1 mL) and **2** (166 mg, 0.10 mmol). The mixture was heated to 80 °C for 24 h. The solvent was removed and the product was dried in vacuo.

^{1}H-NMR (400 MHz, $CDCl_3$) δ = 10.87 (br s, 2H, $(CH_3CH_2)_3N{\cdot}HBr$), 7.76 (s, 4H), 7.67 (d, 4H), 6.98 (d, 4H), 6.83 (s, 4H), 3.12 (q, 12H, $(CH_3CH_2)_3N{\cdot}HBr$), 2.89–2.75 (m 2H), 2.63 (s, 3H, NC*H₃*), 2.50–2.38 (m, 4H), 1.41 (t, 18H, $(CH_3CH_2)_3N{\cdot}HBr$), 1.21 (m, 12H), 0.92 (br d, 12H) ppm.
^{11}B{^{1}H} NMR (128 MHz, $CDCl_3$): δ = 47.9 ppm (s).

3. References

[1] X. Y. Chen, H. Buschmann, C. Bolm, *Synlett* **2012**, *23*, 2808–2810.

[2] K. H. V. Reddy, V. P. Reddy, J. Shankar, B. Madhav, B. S. P. Anil Kumar, Y. V. D. Nageswar, *Tetrahedron Lett.* **2011**, *52*, 2679–2682.

[3] T. Lorenz, M. Crumbach, T. Eckert, A. Lik, H. Helten, *Angew. Chem. Int. Ed.* **2017**, *56*, 2780–2784; *Angew. Chem.* **2017,** *129*, 2824–2828 for the synthesis of the precursor, 1,4-bis(dibromoboryl)benzene, see: M. C. Haberecht, J. B. Heilmann, A. Haghiri, M. Bolte, J. W. Bats, H.-W. Lerner, M. C. Holthausen, M. Wagner, *Z. Anorg. Allg. Chem.* **2004**, *630*, 904–913.

4. NMR spectra

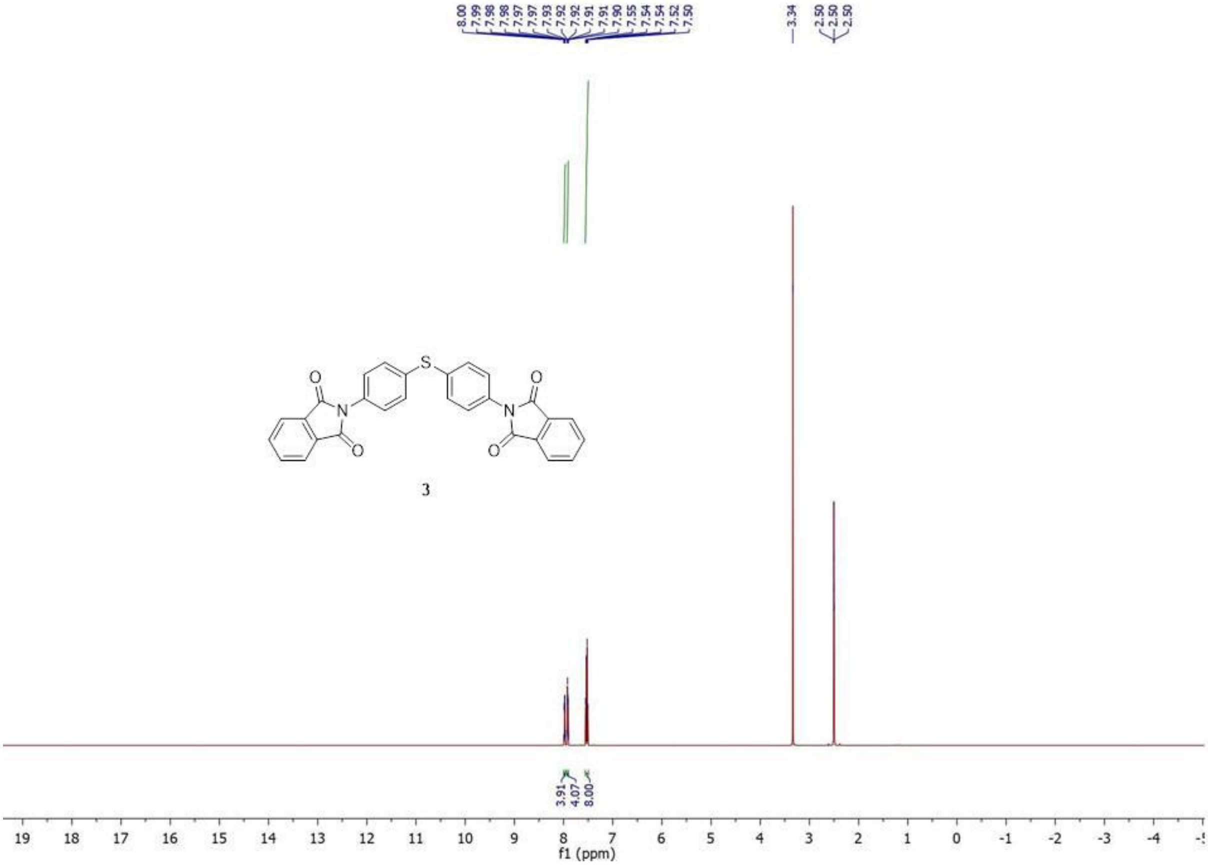

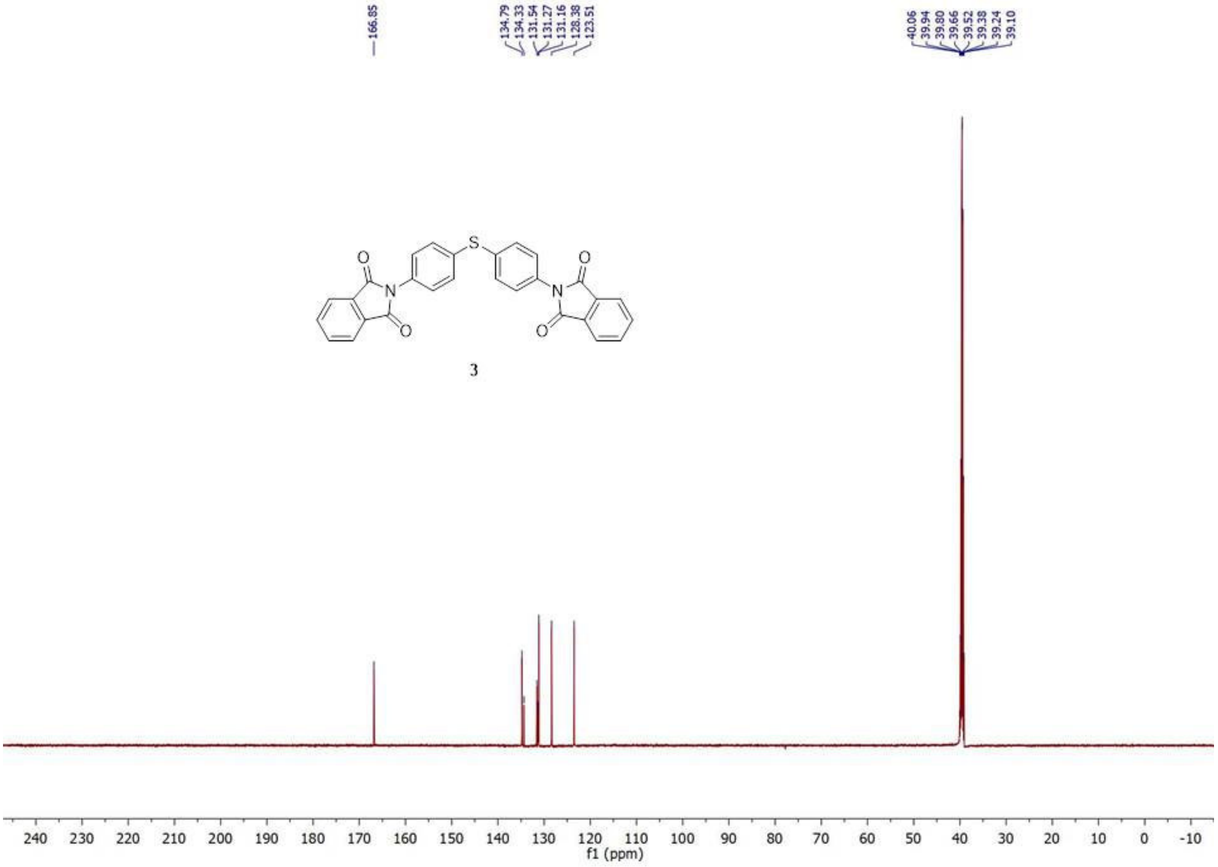

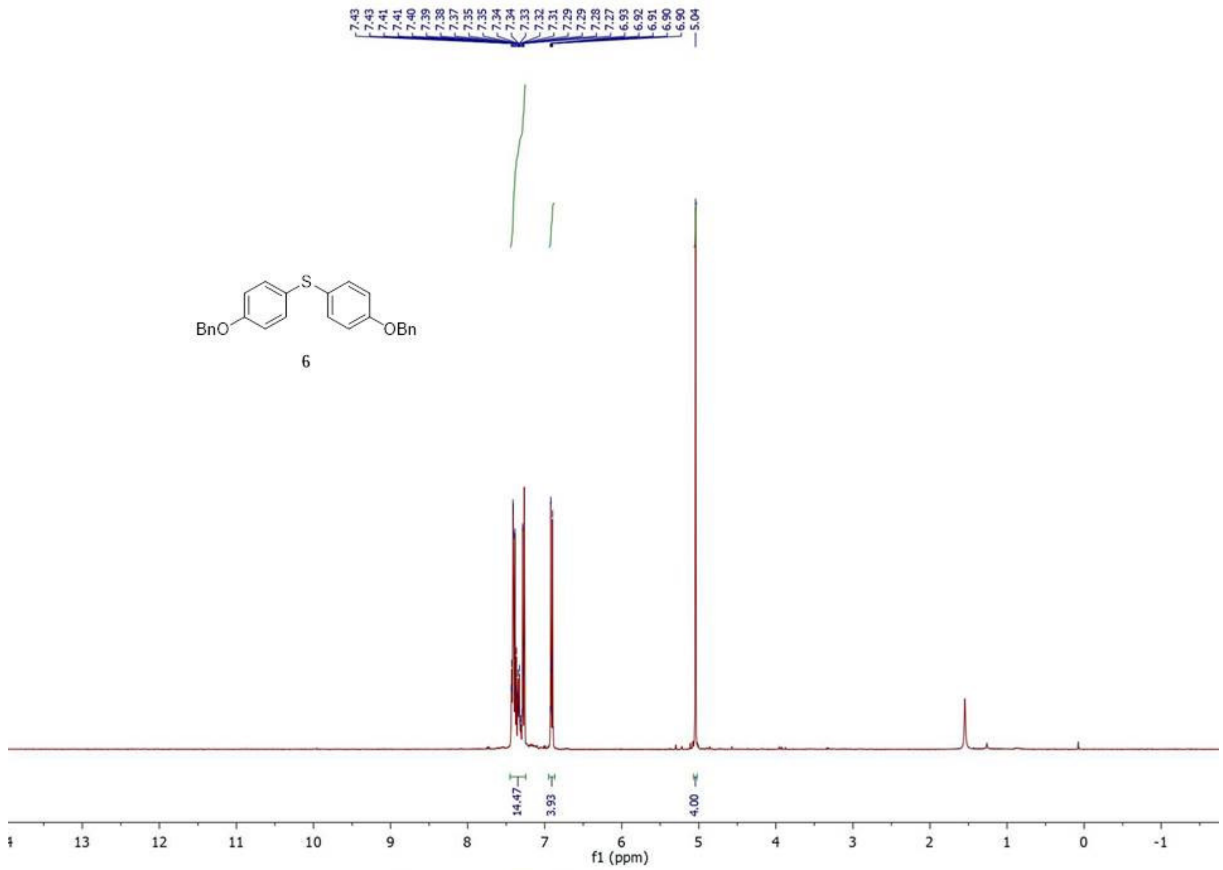
7.43
7.43
7.41
7.40
7.39
7.38
7.37
7.35
7.35
7.34
7.34
7.33
7.32
7.31
7.29
7.29
7.28
7.27
6.93
6.92
6.91
6.90
6.90
5.04
S
BnO
OBn
6
14.47
3.93
4.00
f1 (ppm)

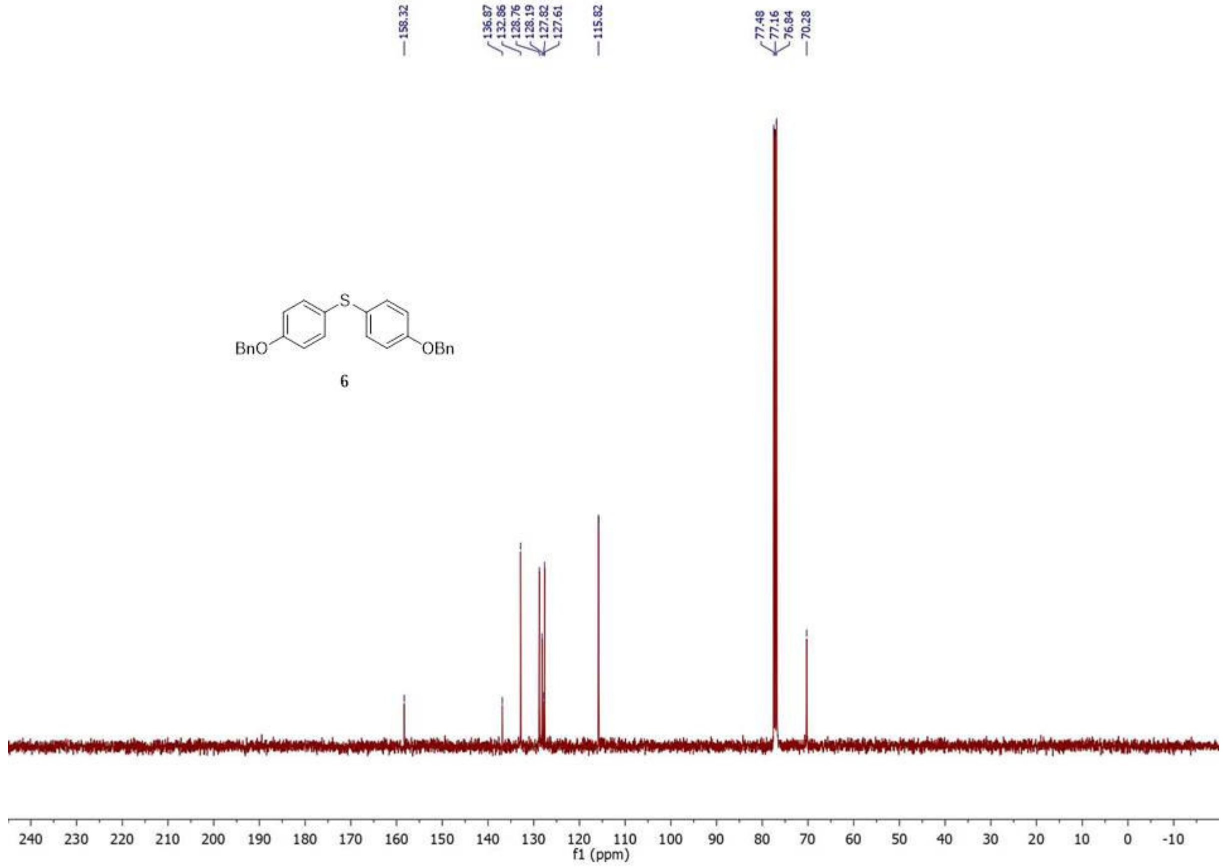
158.32
136.87
132.86
128.76
128.19
127.82
127.61
115.82
77.48
77.16
76.84
70.28
S
BnO
OBn
6
f1 (ppm)

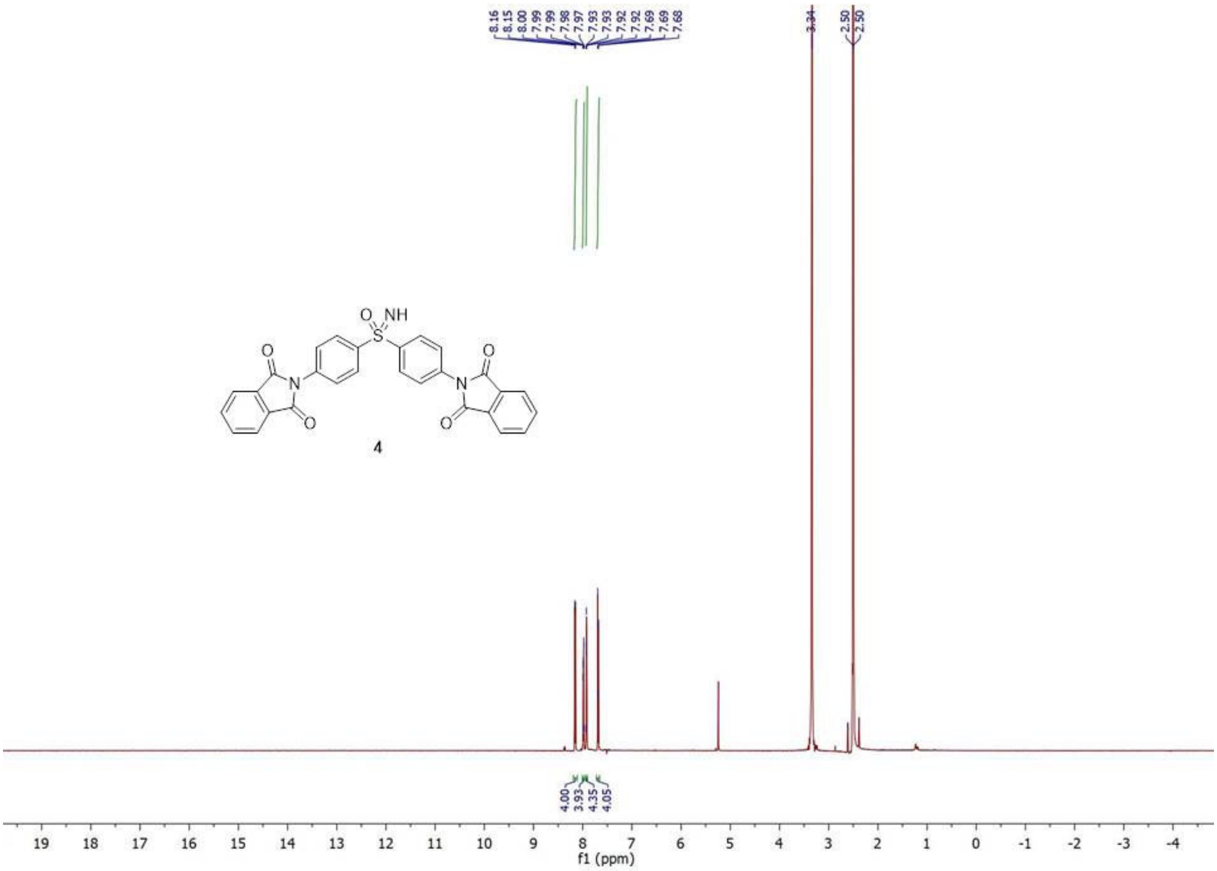
8.16
8.15
8.00
7.99
7.99
7.98
7.97
7.93
7.93
7.92
7.92
7.69
7.68
3.34
2.50
2.50
4
4.00
3.93
4.35
4.05
f1 (ppm)

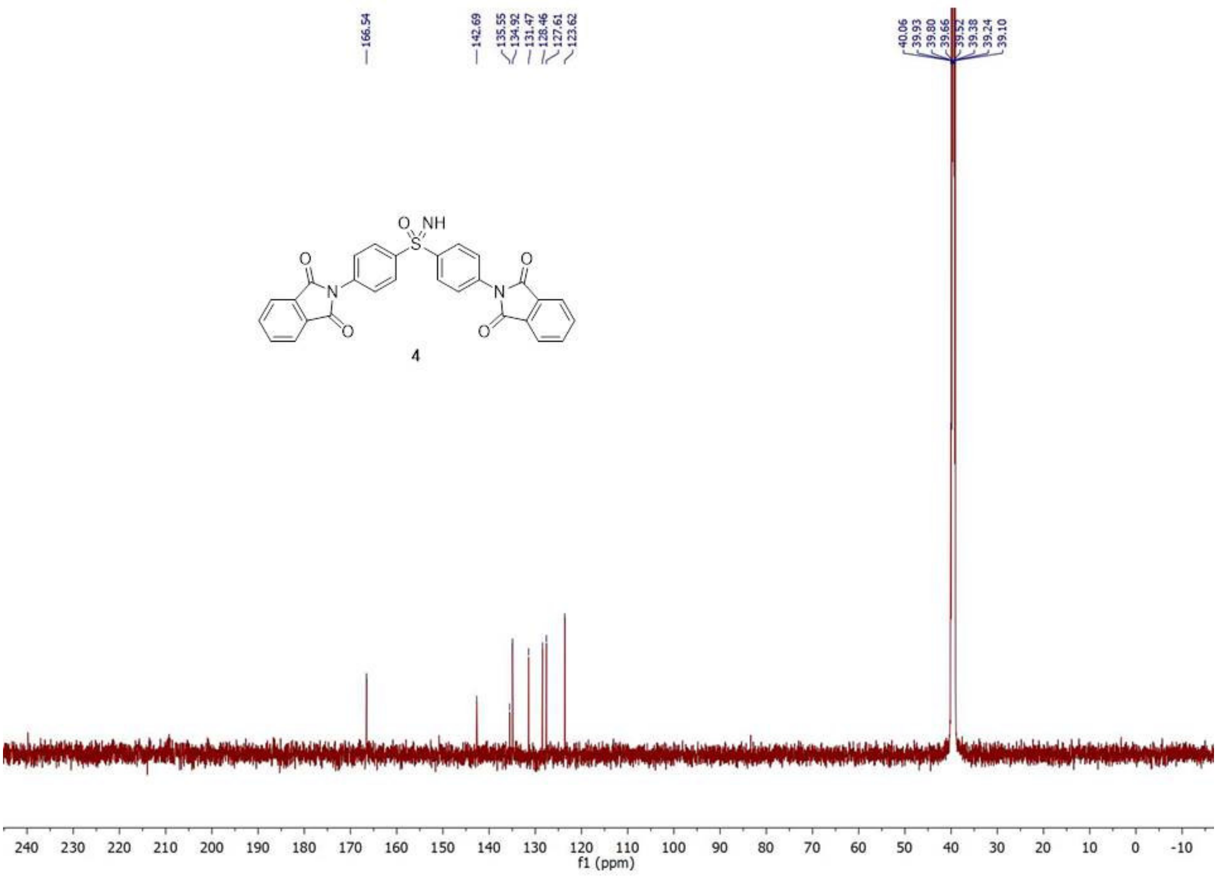
166.54
142.69
135.55
134.92
131.47
128.46
127.61
123.62
40.06
39.93
39.80
39.66
39.52
39.38
39.24
39.10
4
f1 (ppm)

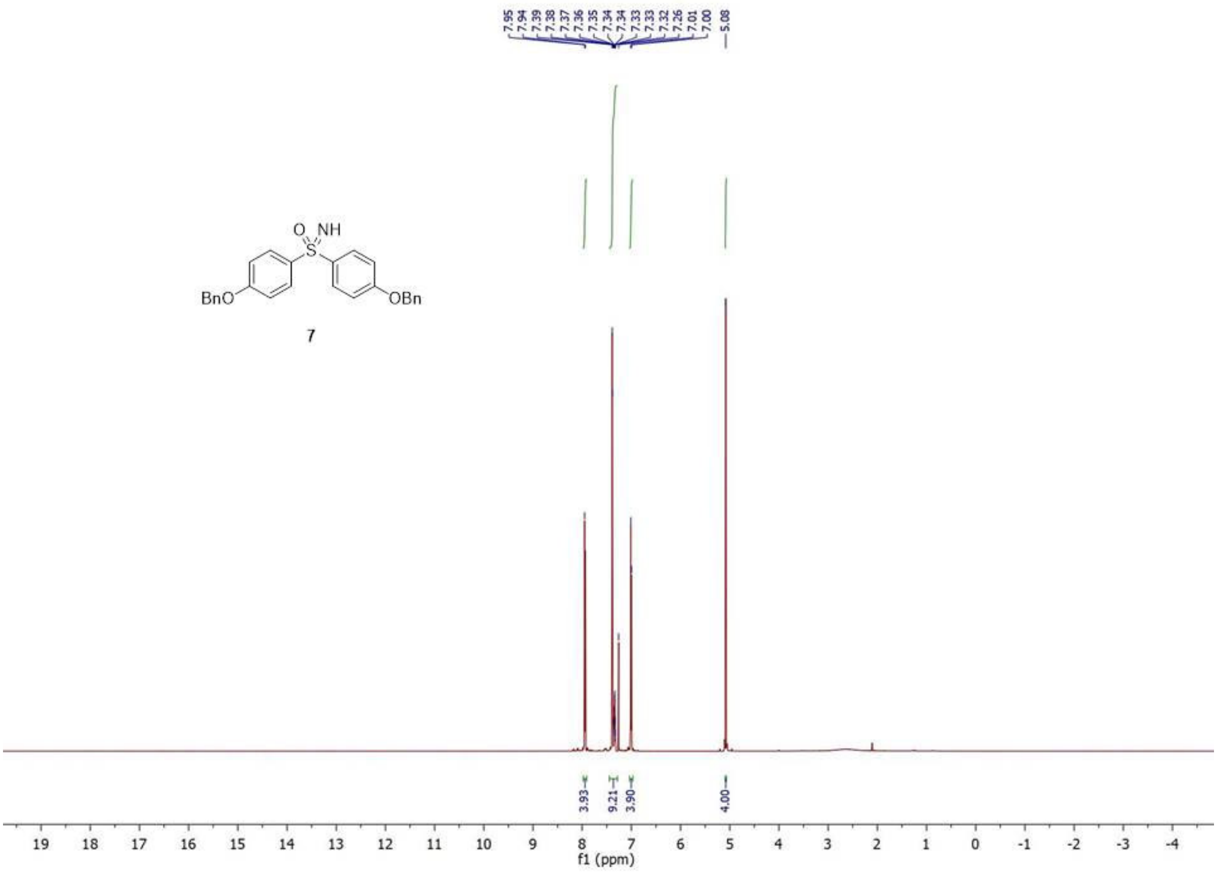
7.95
7.94
7.39
7.38
7.37
7.36
7.35
7.34
7.34
7.33
7.33
7.32
7.26
7.01
7.00
5.08
O NH
S
BnO
OBn
7
3.93
9.21
3.90
4.00
f1 (ppm)

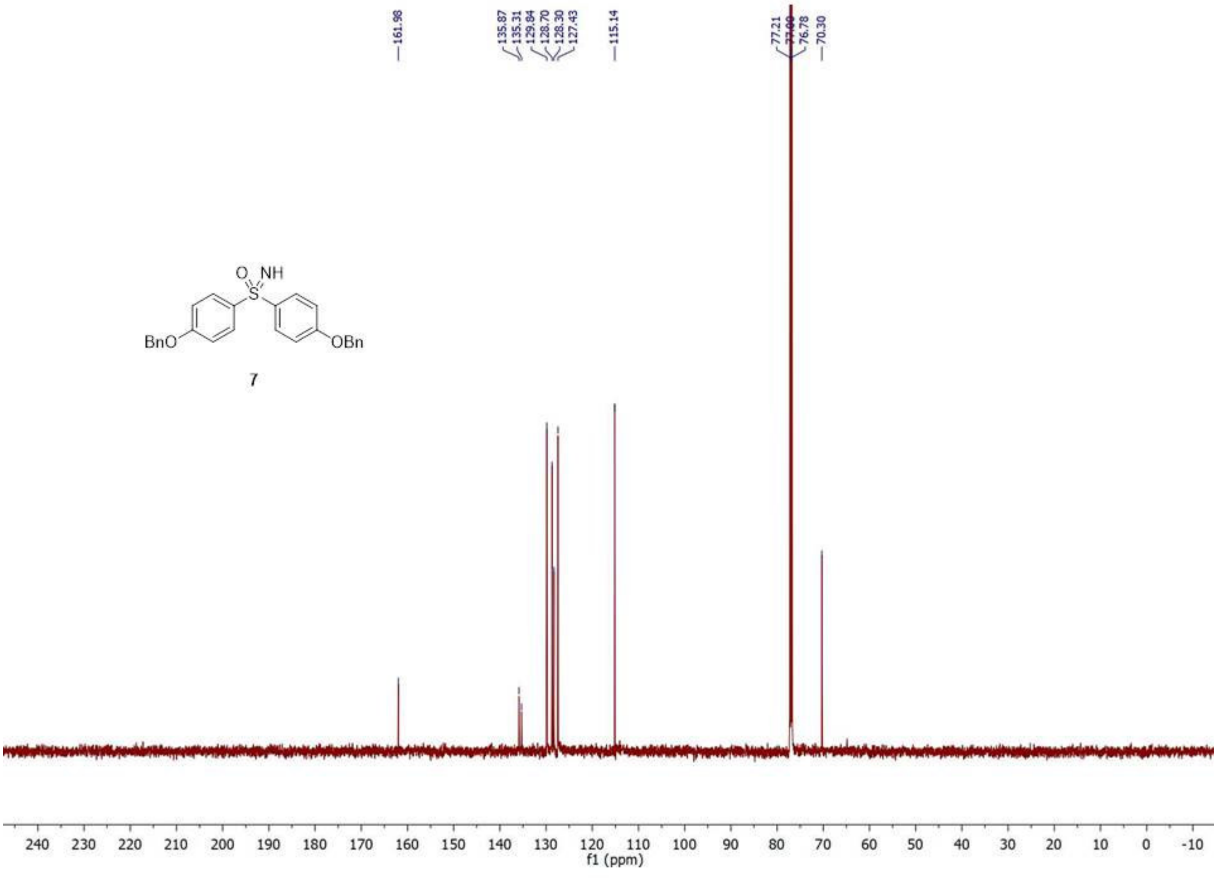
161.98
135.87
135.31
129.84
128.70
128.30
127.43
115.14
77.21
76.78
70.30
O NH
S
BnO
OBn
7
f1 (ppm)

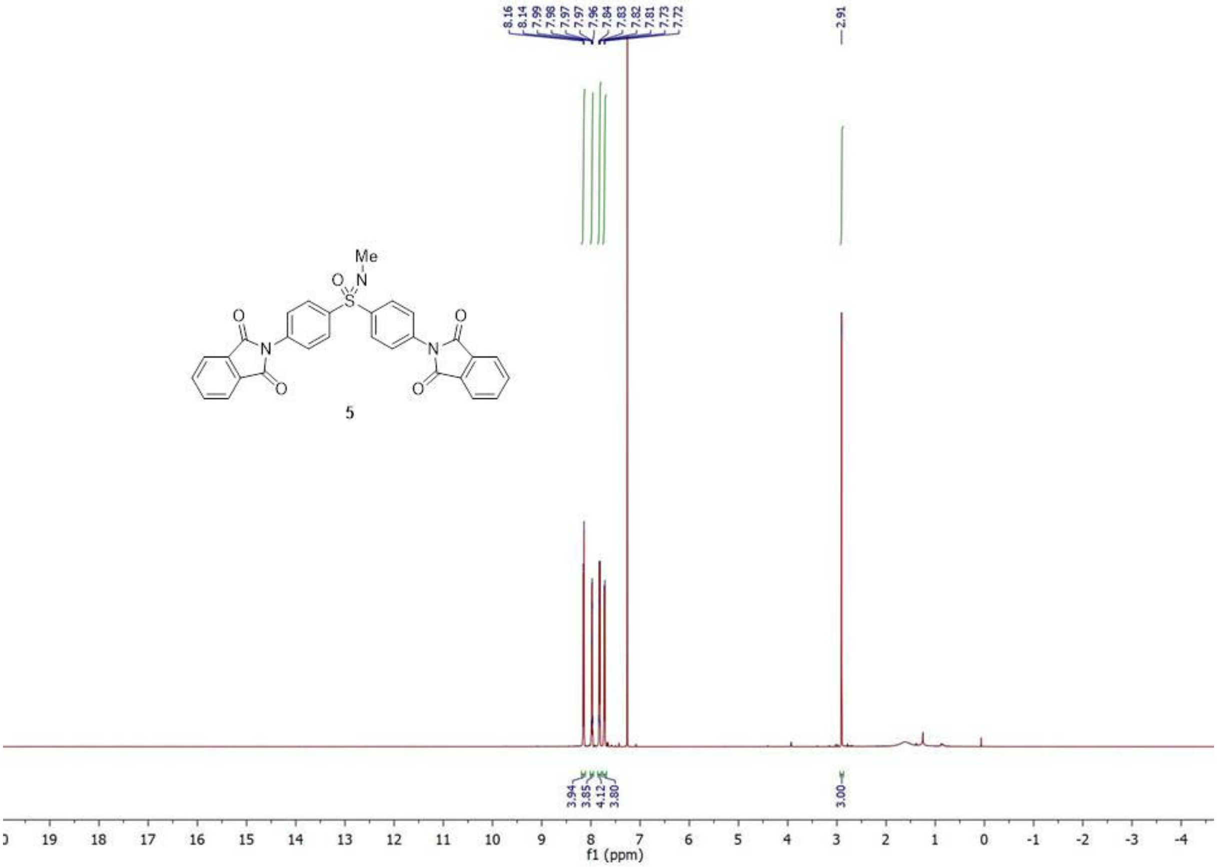
8.16
8.14
7.99
7.98
7.97
7.97
7.96
7.84
7.83
7.82
7.81
7.73
7.72
2.91
Me
N
O
S
O
N
O
O
N
O
3.94
3.85
4.12
3.80
3.00
f1 (ppm)

5

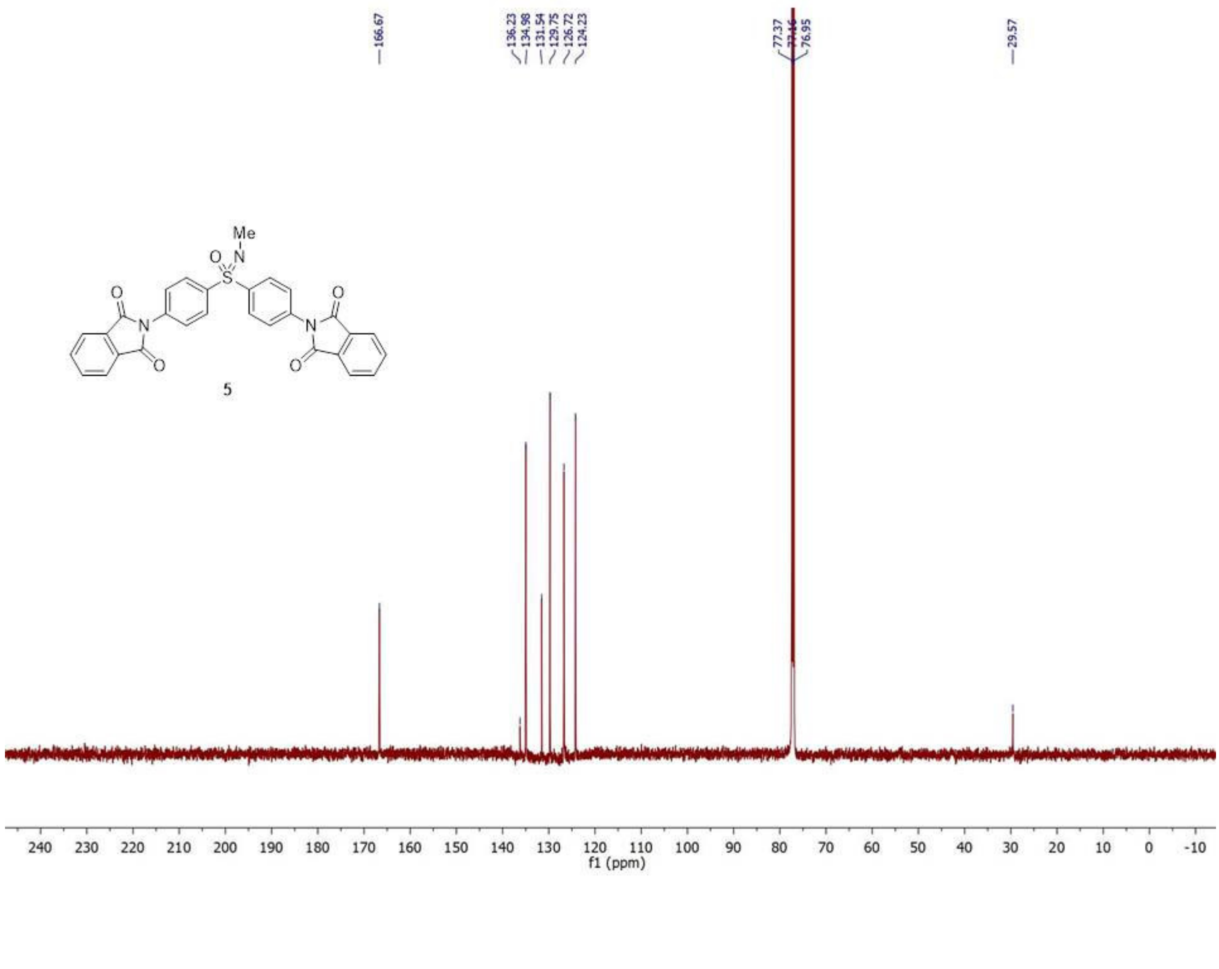
166.67
136.23
134.98
131.54
129.75
126.72
124.23
77.37
77.16
76.95
29.57
Me
N
O
S
O
N
O
O
N
O
f1 (ppm)

5

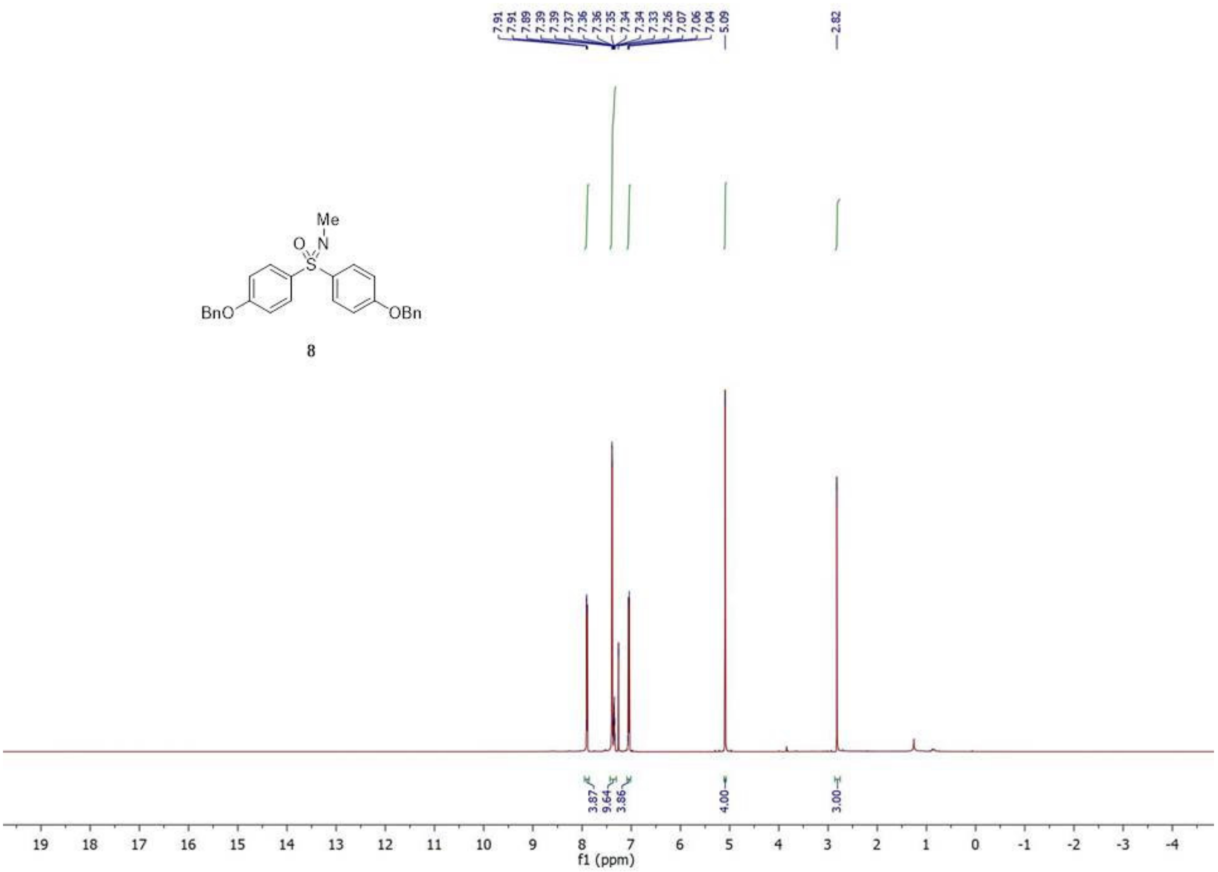
7.91
7.91
7.89
7.39
7.39
7.37
7.36
7.36
7.35
7.34
7.34
7.33
7.26
7.07
7.06
7.04
5.09
2.82
Me
N
O
S
BnO
OBn
8
3.87
9.64
3.86
4.00
3.00
19 18 17 16 15 14 13 12 11 10 9 8 7 6 5 4 3 2 1 0 -1 -2 -3 -4
f1 (ppm)

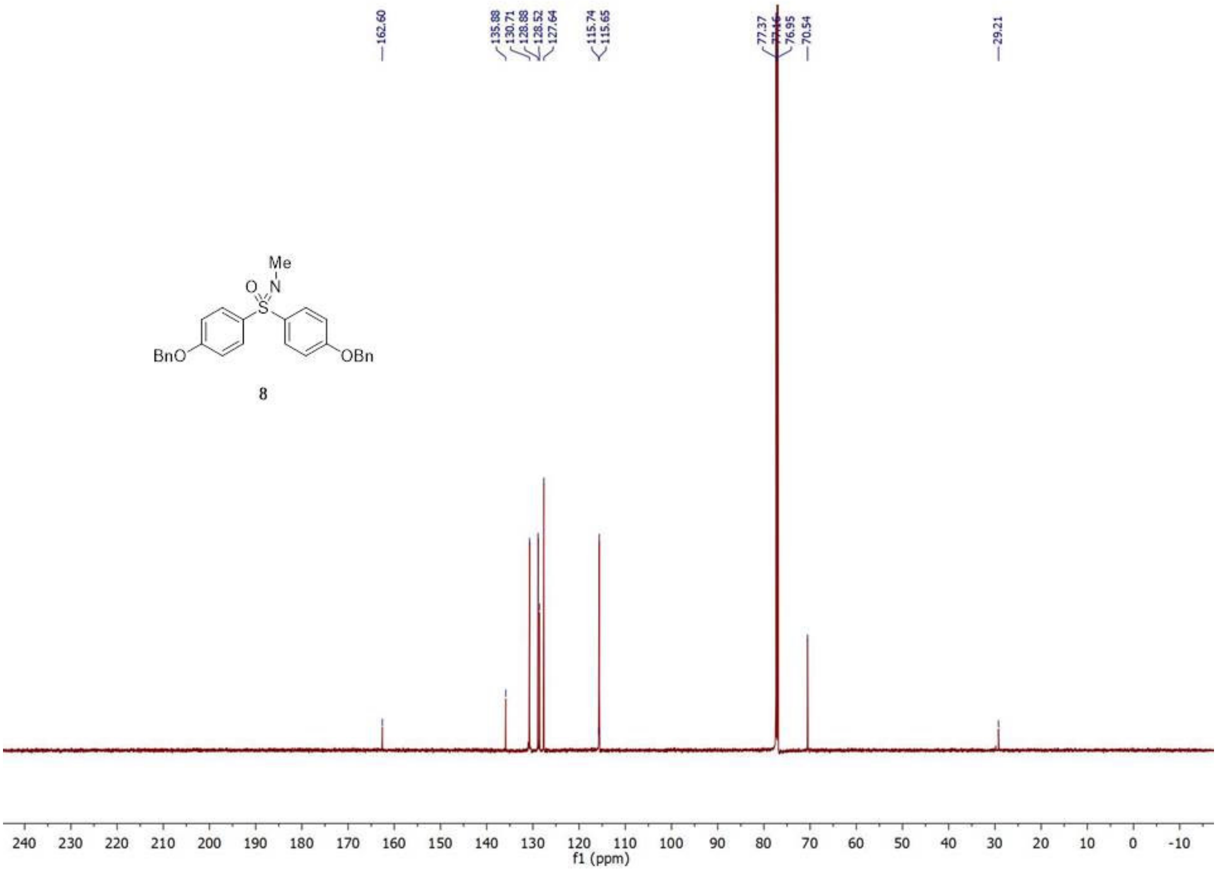
162.60
135.88
130.71
128.88
128.52
127.64
115.74
115.65
77.37
77.16
76.95
70.54
29.21
Me
N
O
S
BnO
OBn
8
240 230 220 210 200 190 180 170 160 150 140 130 120 110 100 90 80 70 60 50 40 30 20 10 0 -10
f1 (ppm)

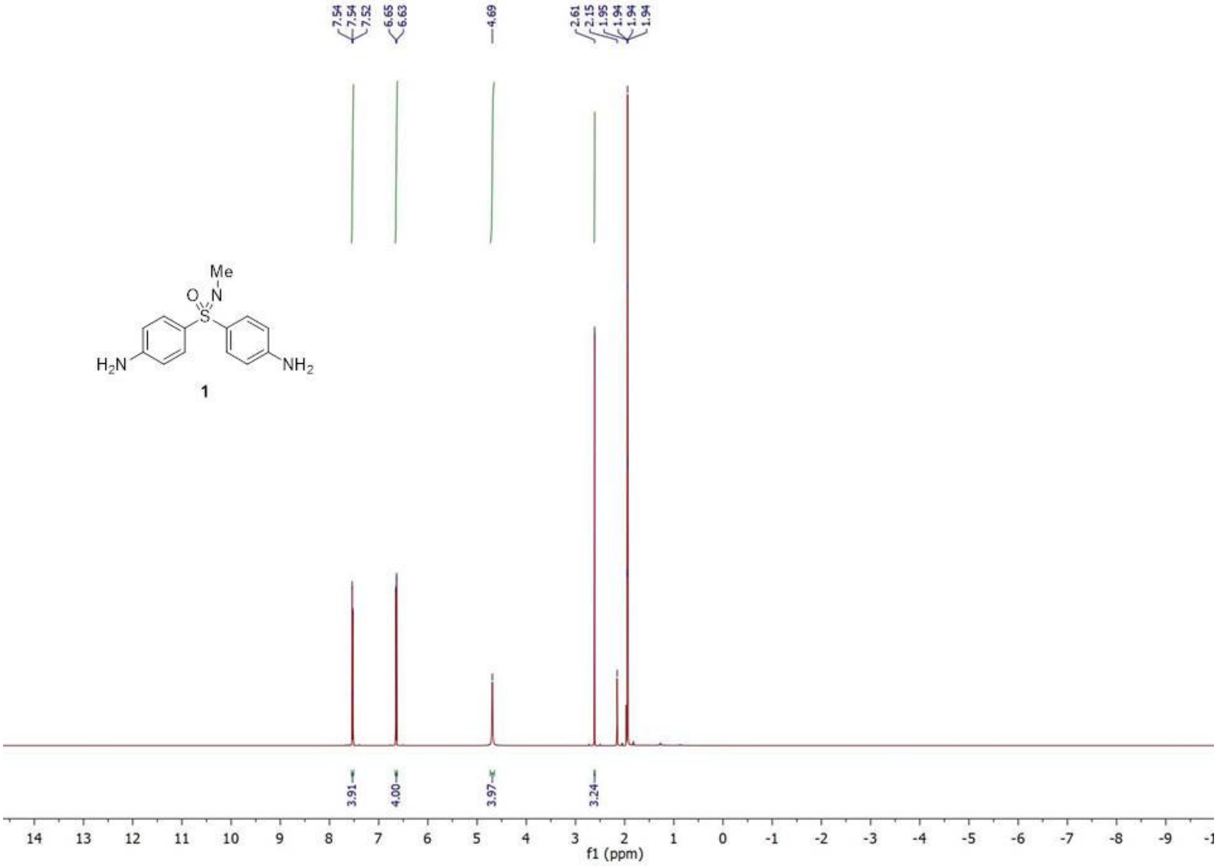
7.54
7.54
7.52
6.65
6.63
4.69
2.61
2.15
1.95
1.94
1.94
1.94
Me
N
O
S
H2N
NH2
1
3.91
4.00
3.97
3.24
f1 (ppm)

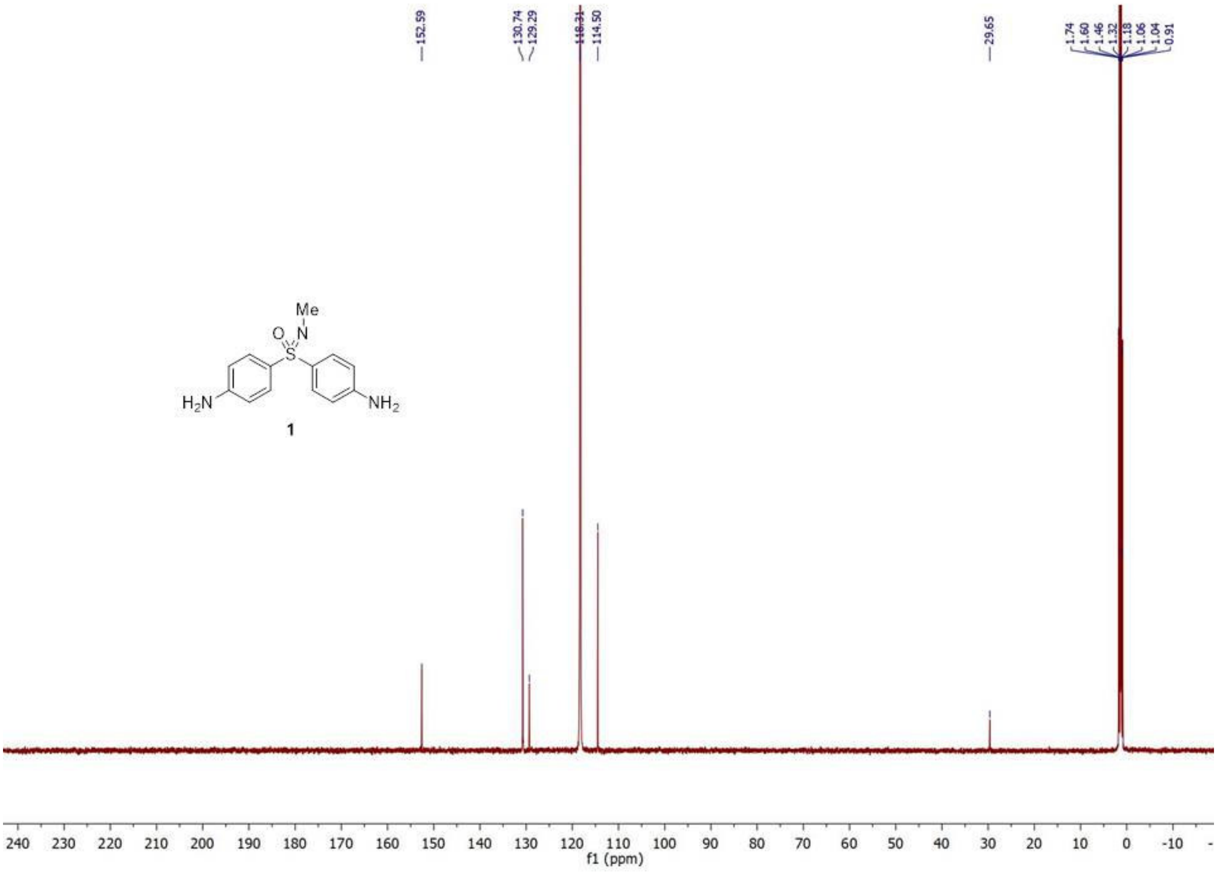
152.59
130.74
129.29
118.31
114.50
29.65
1.74
1.60
1.46
1.32
1.18
1.06
1.04
0.91
Me
N
O
S
H2N
NH2
1
f1 (ppm)

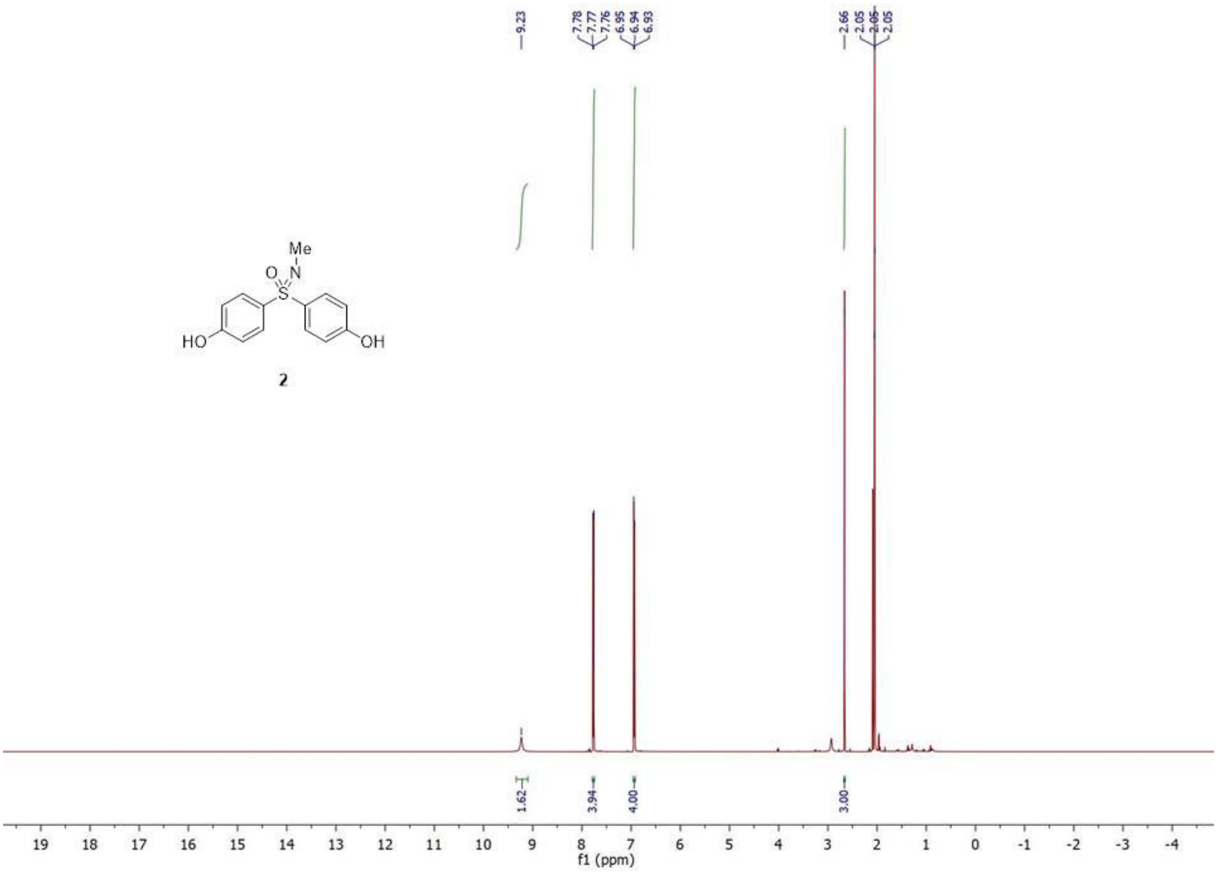
9.23
7.78
7.77
7.76
6.95
6.94
6.93
2.66
2.05
2.05
2.05
Me
N
O
S
HO
OH
2
1.62
3.94
4.00
3.00
19 18 17 16 15 14 13 12 11 10 9 8 7 6 5 4 3 2 1 0 -1 -2 -3 -4
f1 (ppm)

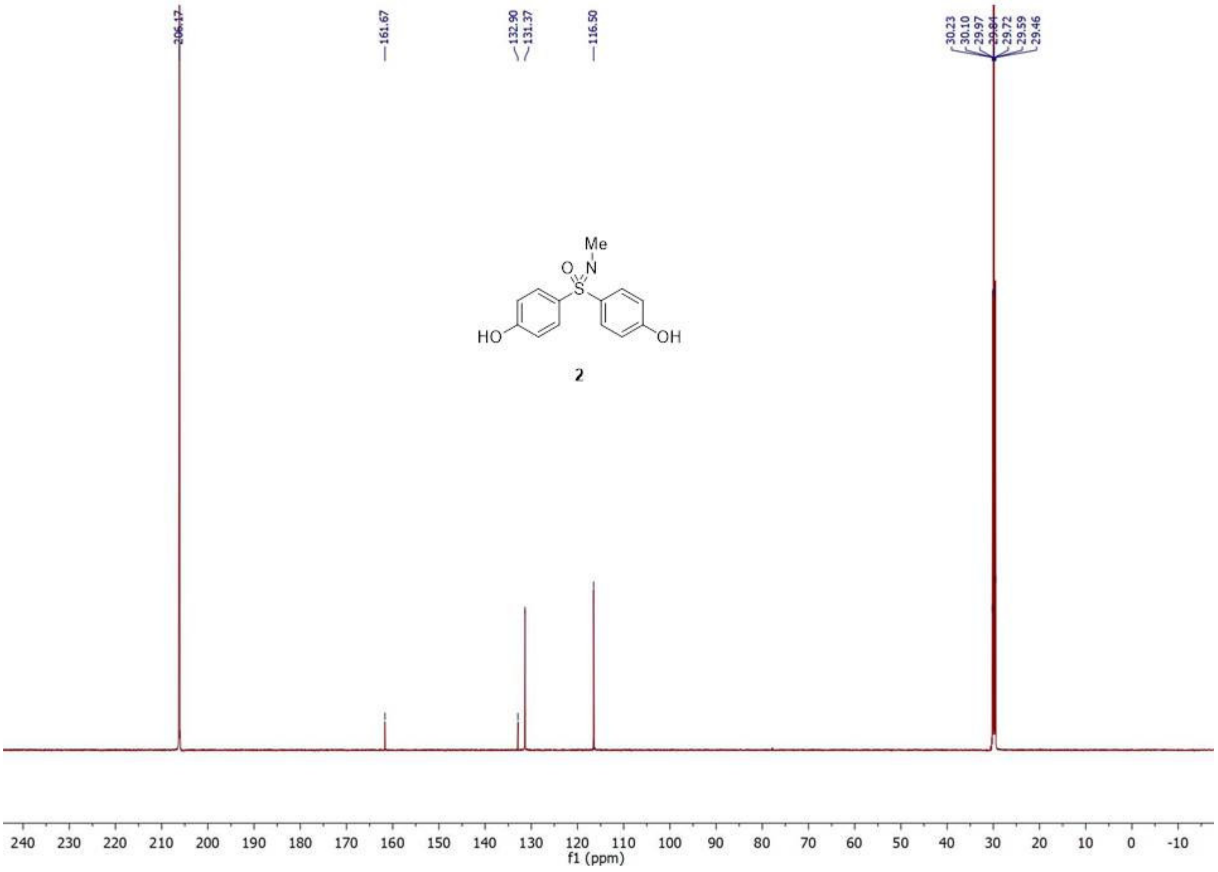
206.17
161.67
132.90
131.37
116.50
30.23
30.10
29.97
29.84
29.72
29.59
29.46
Me
N
O
S
HO
OH
2
240 230 220 210 200 190 180 170 160 150 140 130 120 110 100 90 80 70 60 50 40 30 20 10 0 -10
f1 (ppm)

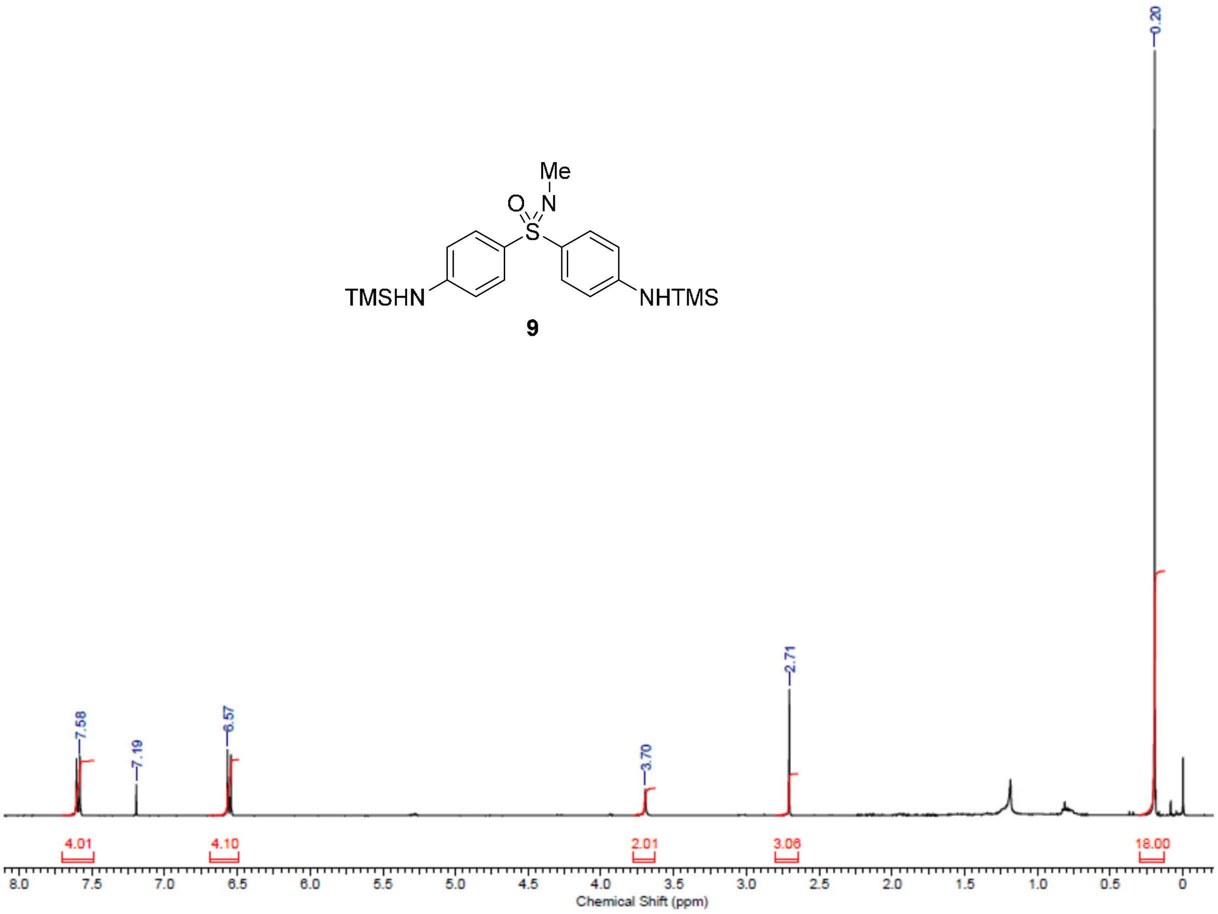
Me
N
O
S
TMSHN
NHTMS
9
7.58
7.19
6.57
3.70
2.71
0.20
4.01
4.10
2.01
3.06
18.00
Chemical Shift (ppm)

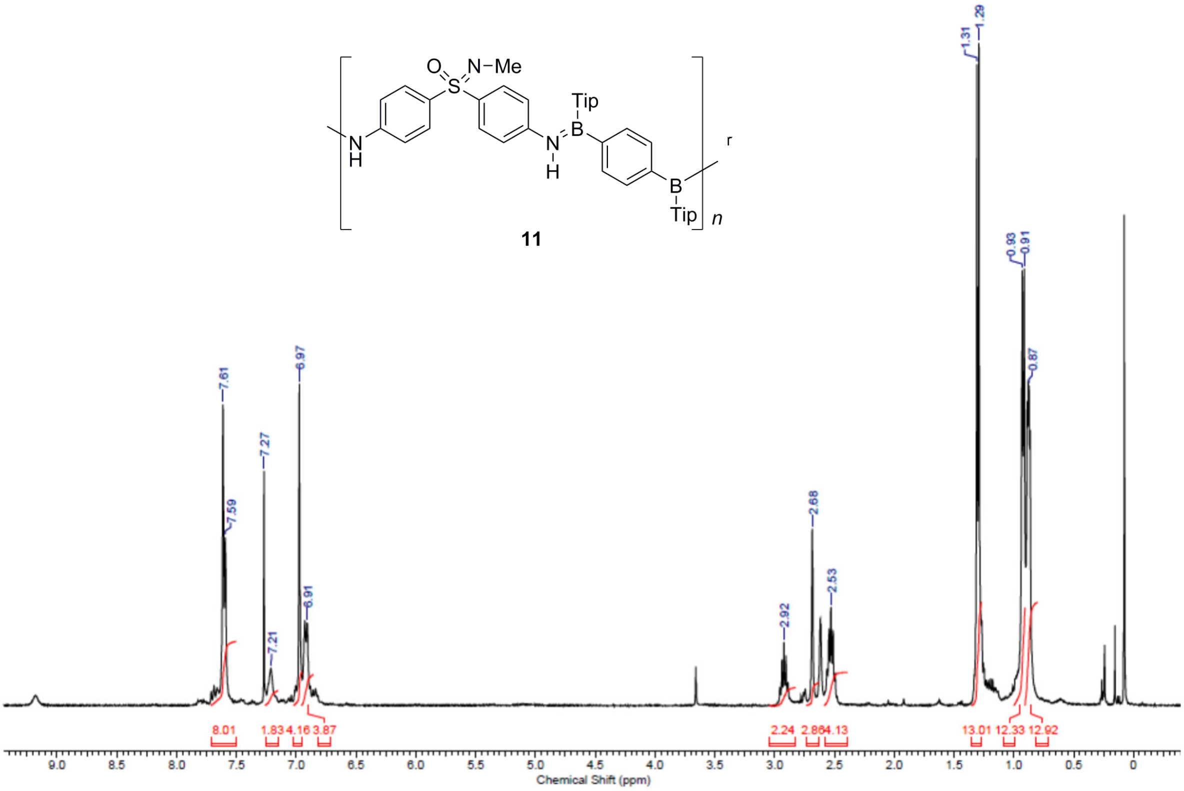
O
S
N–Me
Tip
B
N
H
B
Tip
n
11
7.61
7.59
7.27
7.21
6.97
6.91
2.92
2.68
2.53
1.31
1.29
0.93
0.91
0.87
8.01
1.83
4.16
3.87
2.24
2.86
4.13
13.01
12.33
12.92
Chemical Shift (ppm)

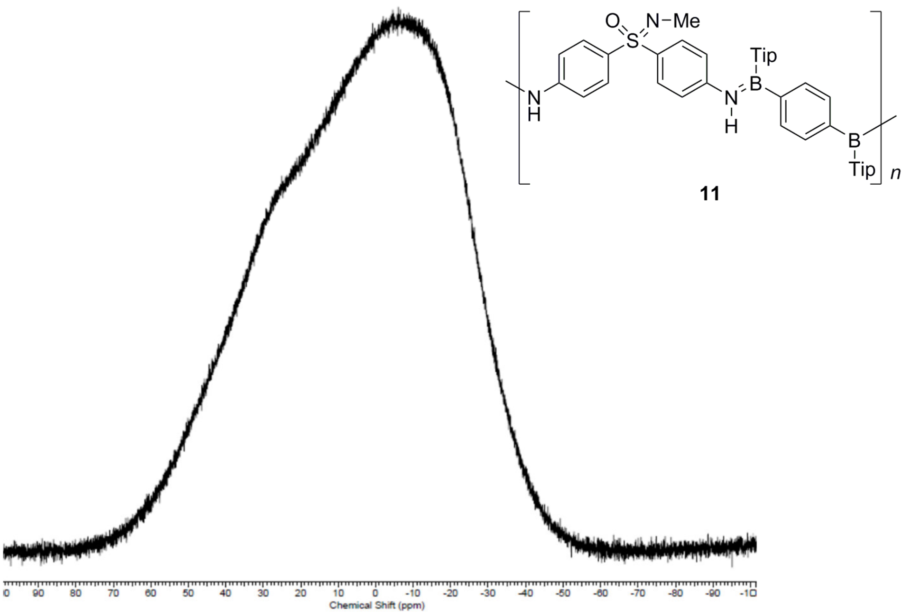

$^{11}B\{^{1}H\}$ NMR spectrum of **11** (in $CDCl_3$). No signal for **11** could be detected; only the glass background is seen.

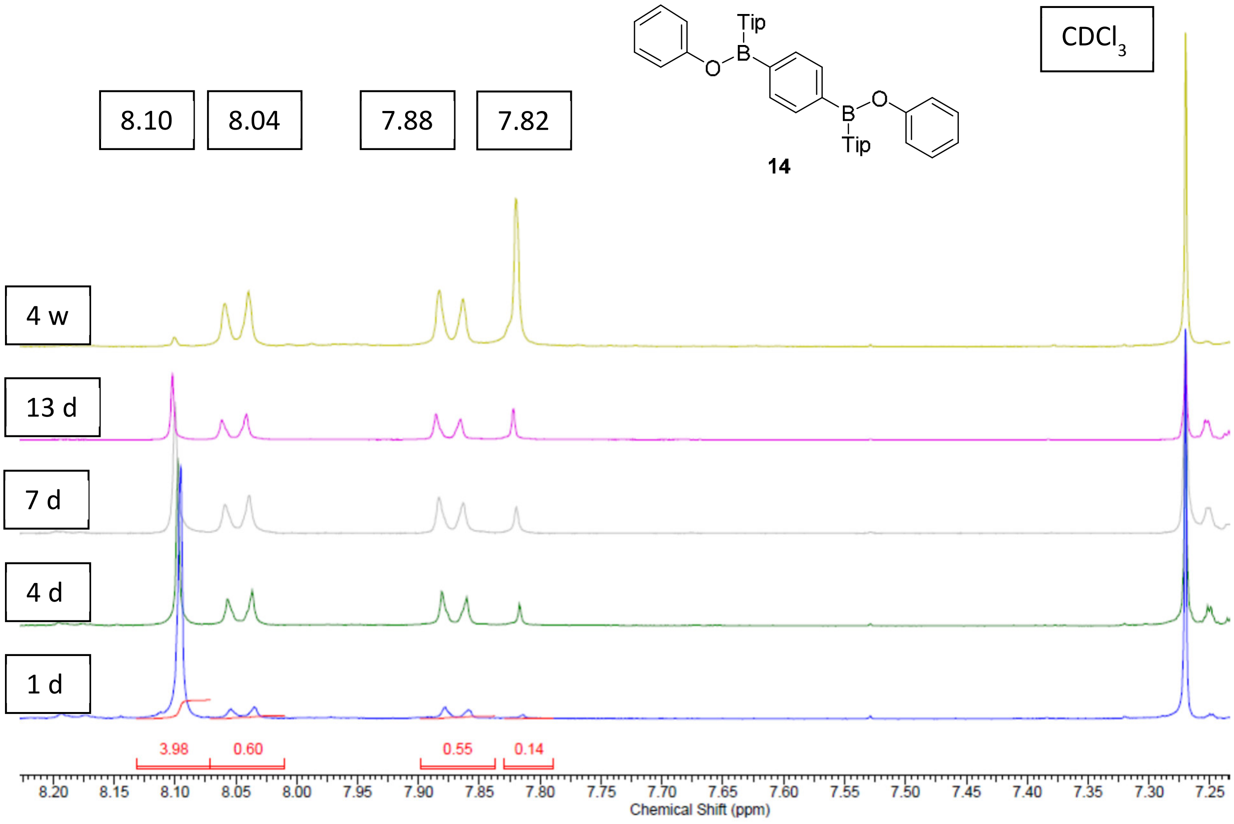

Detail of ^{1}H-NMR spectra (in $CDCl_3$) recorded during the attempted synthesis of **14** from **10** and **12** in DCM at room temperature. The resonance at 8.10 ppm comes from the reactant **10**, that at 7.81 ppm is assigned to **14**, and the signals at 8.04 and 7.88 ppm are tentatively assigned to the an intermediate in which only one boron site of **10** had reacted (i.e., protons of the phenylene group bridging the two boron centers in each case).

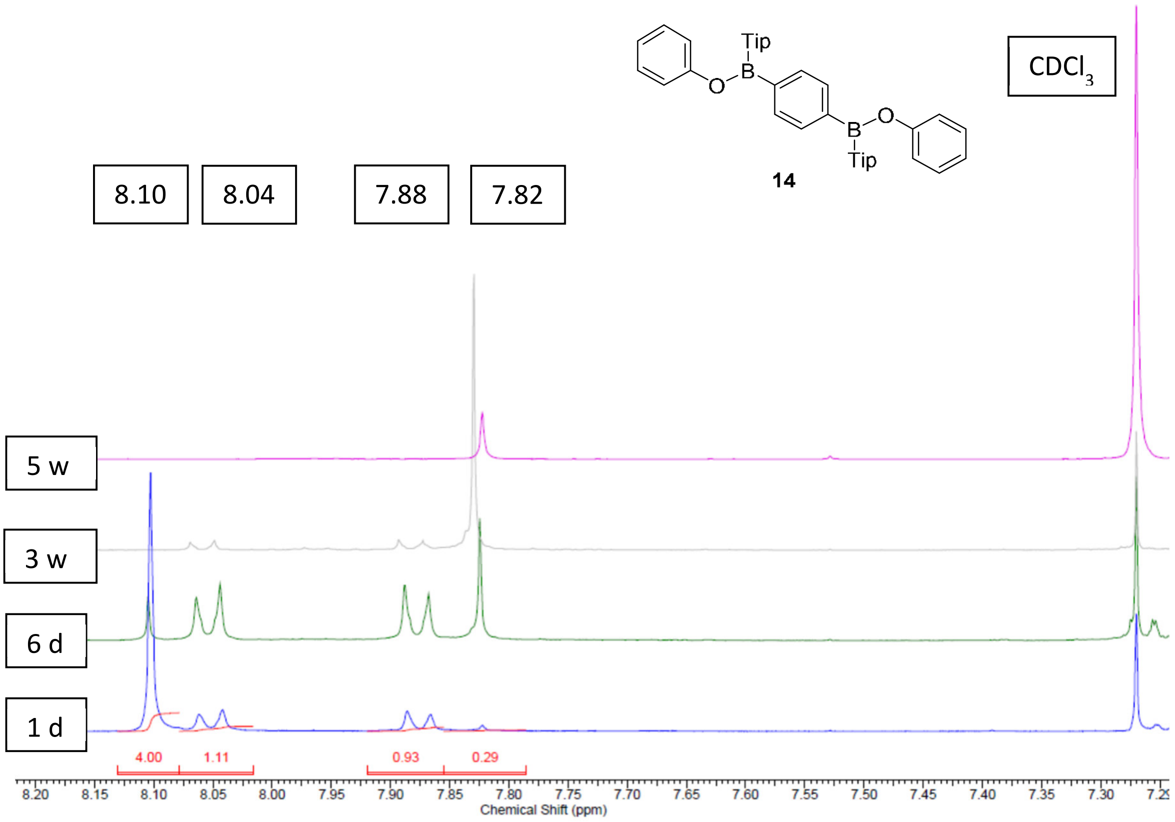

Detail of ^{1}H-NMR spectra (in $CDCl_3$) recorded during the attempted synthesis of **14** from **10** and **12** in *o*-DFB at 80 °C. The resonance at 8.10 ppm comes from the reactant **10**, that at 7.82 ppm is assigned to **14**, and the signals at 8.04 and 7.88 ppm are tentatively assigned to the an intermediate in which only one boron site of **10** had reacted (i.e., protons of the phenylene group bridging the two boron centers in each case). In the spectrum recorded after 5 weeks the intensity of the product signal is low because for this aliquot a larger excess of $CDCl_3$ was used in this case.

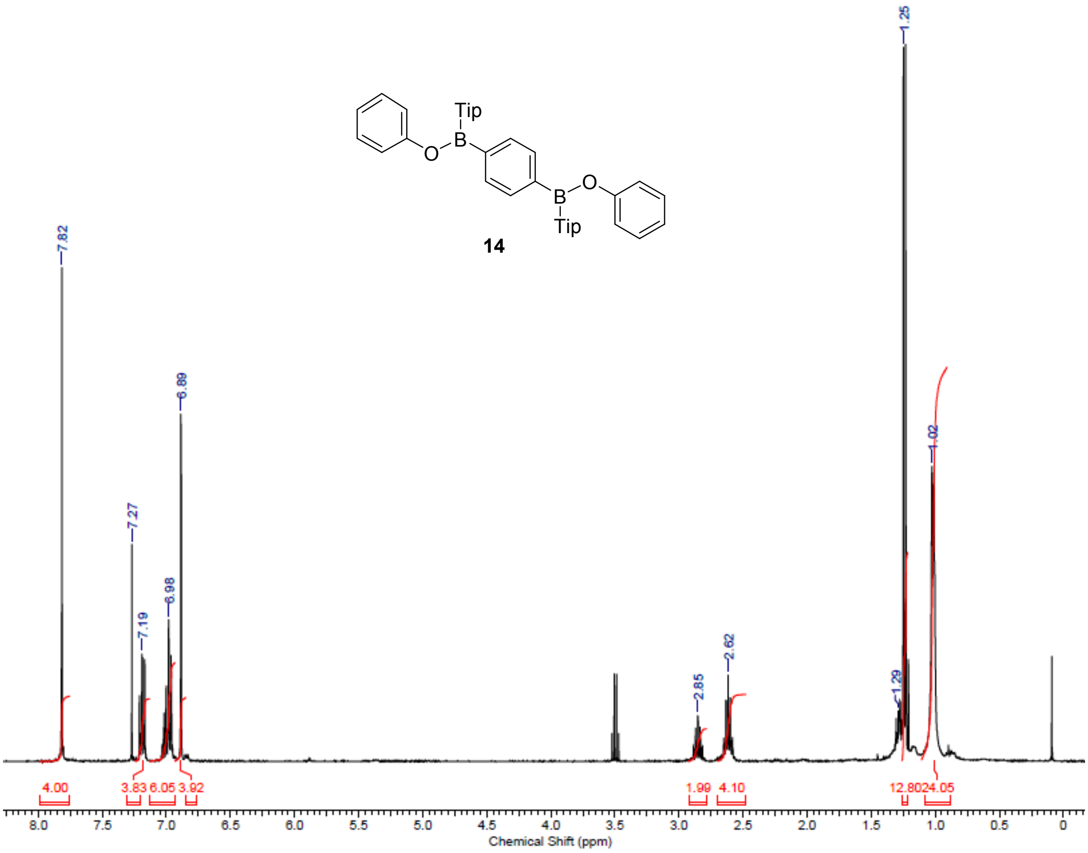
Tip
O
B
B
O
Tip
14
7.82
7.27
7.19
6.98
6.89
2.85
2.62
1.29
1.25
1.02
4.00
3.83
6.05
3.92
1.99
4.10
12.80
24.05
8.0 7.5 7.0 6.5 6.0 5.5 5.0 4.5 4.0 3.5 3.0 2.5 2.0 1.5 1.0 0.5 0
Chemical Shift (ppm)

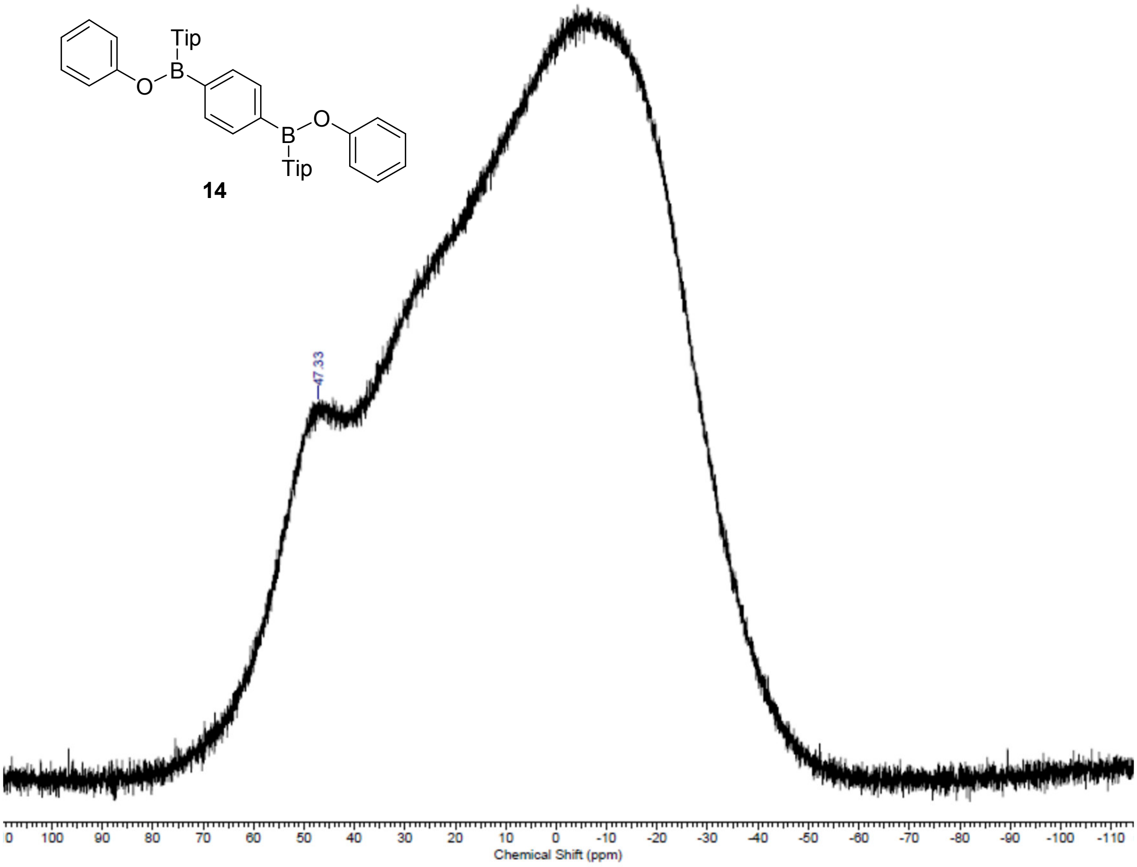
Tip
O
B
B
O
Tip
14
47.33
100 90 80 70 60 50 40 30 20 10 0 -10 -20 -30 -40 -50 -60 -70 -80 -90 -100 -110
Chemical Shift (ppm)

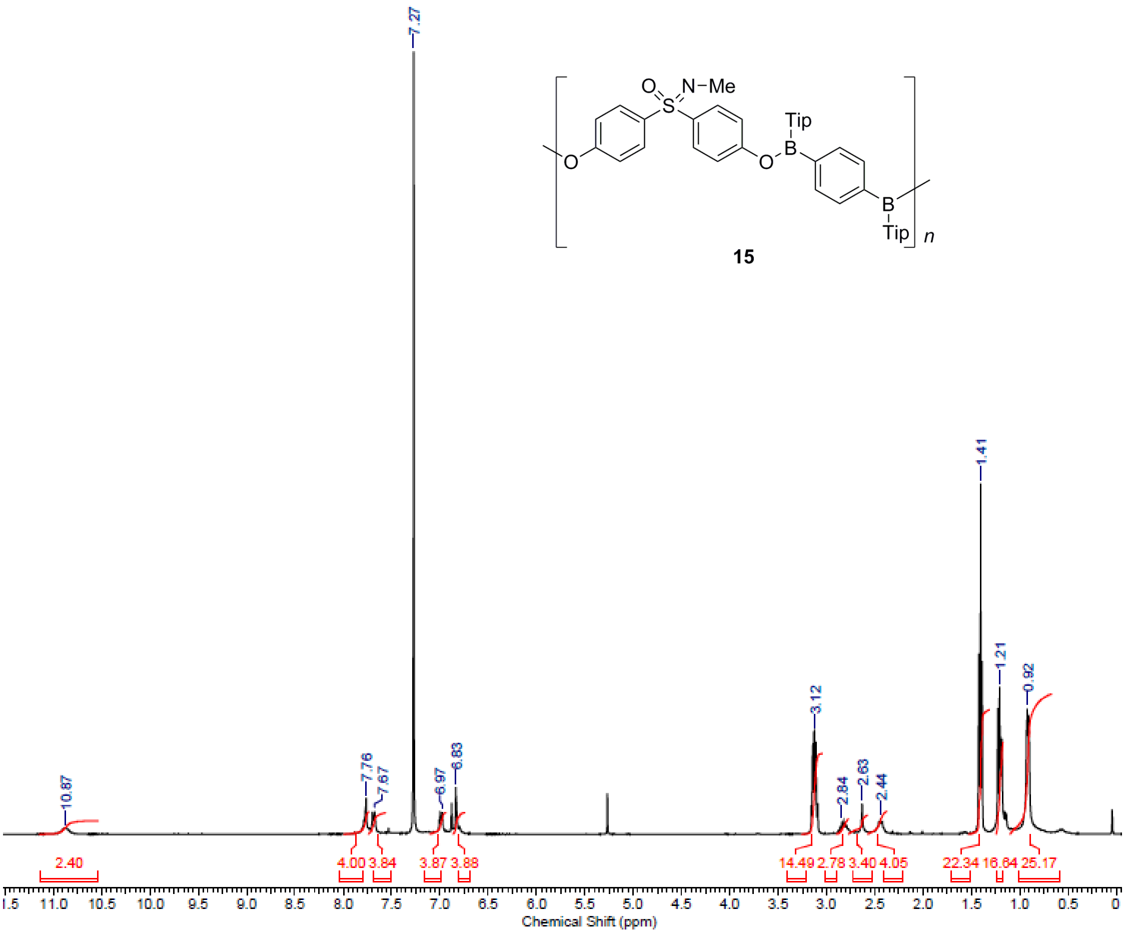
O
N−Me
S
O
Tip
B
O
B
Tip
n
15
7.27
10.87
7.76
7.67
6.97
6.83
3.12
2.84
2.63
2.44
1.41
1.21
0.92
2.40
4.00
3.84
3.87
3.88
14.49
2.78
3.40
4.05
22.34
16.64
25.17
Chemical Shift (ppm)

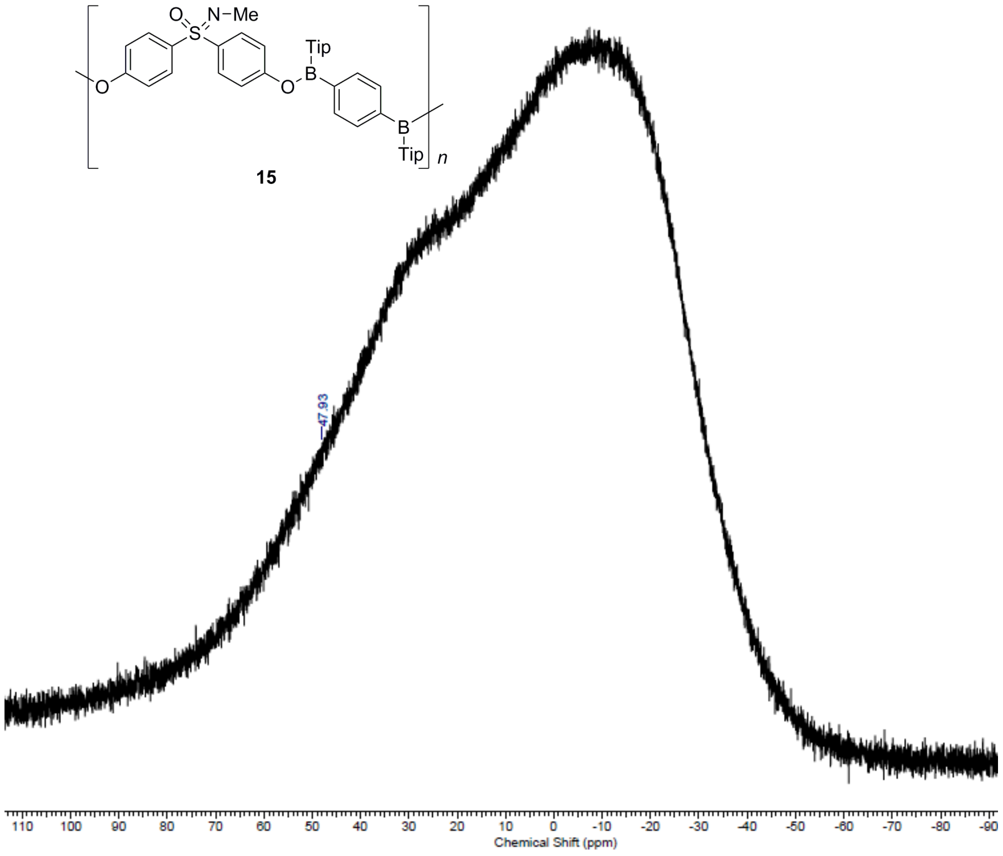
O
N−Me
S
O
Tip
B
O
B
Tip
n
15
47.93
Chemical Shift (ppm)

4. GPC traces

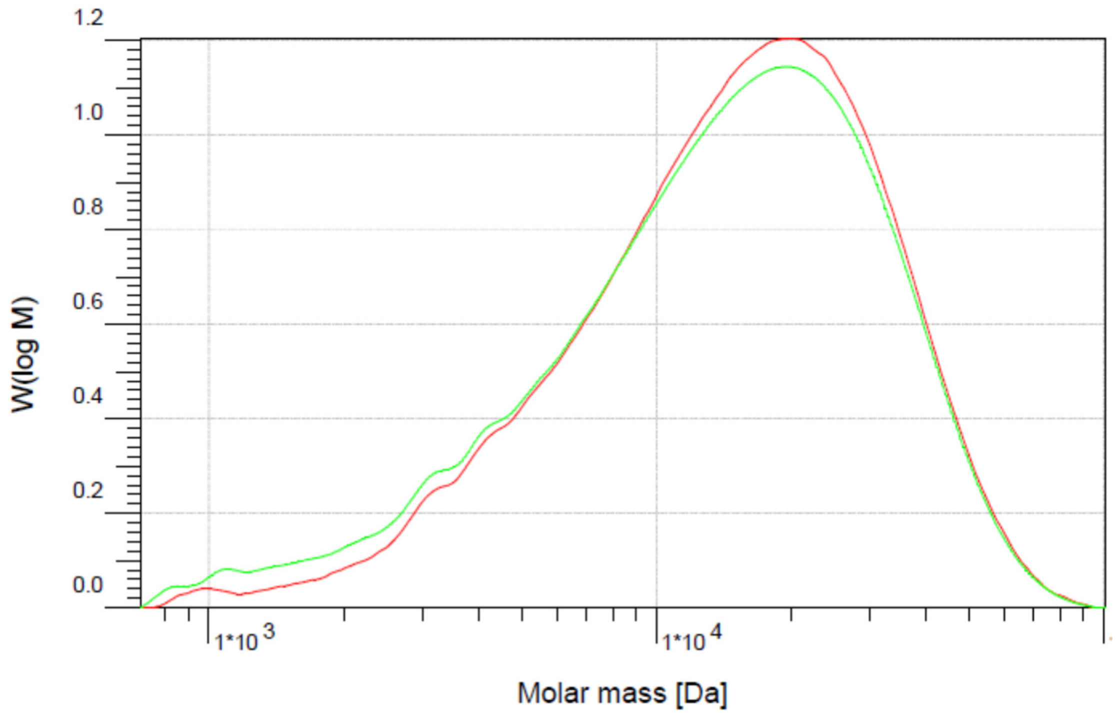

Gel permeation chromatography (GPC) trace of **11** (trial 1) (in THF); red line: detection by RI signal; green line: detection by UV signal.

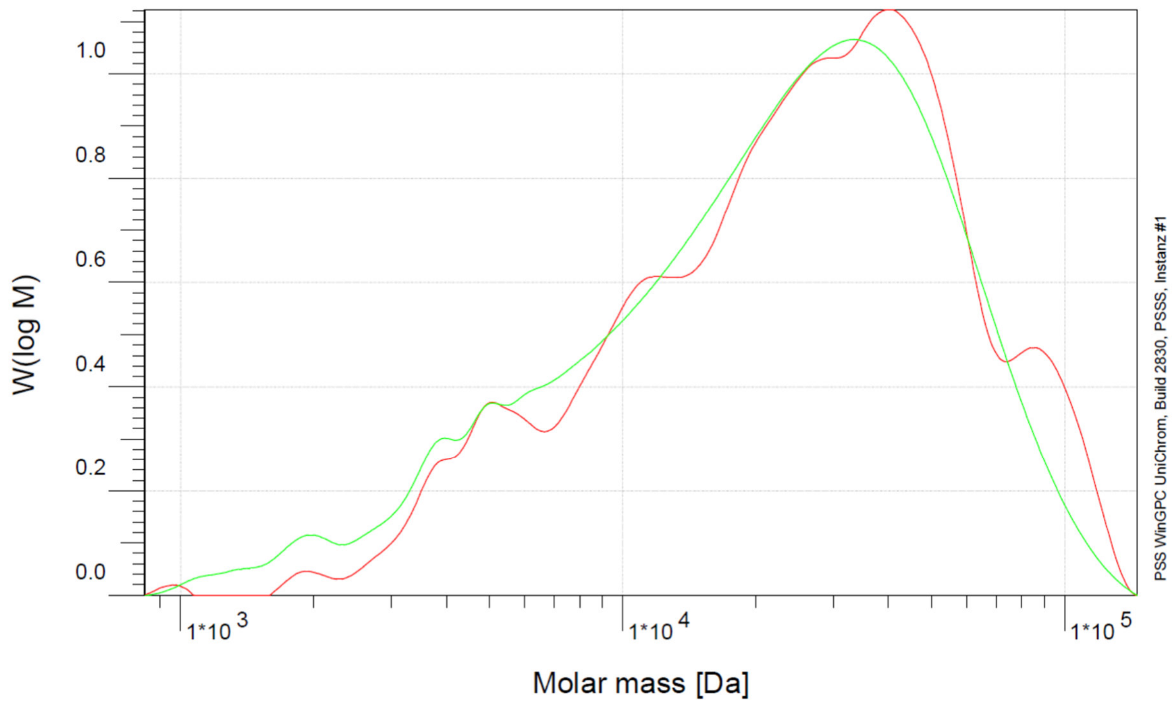

Gel permeation chromatography (GPC) trace of **11** (trial 2) (in THF); red line: detection by RI signal; green line: detection by UV signal.

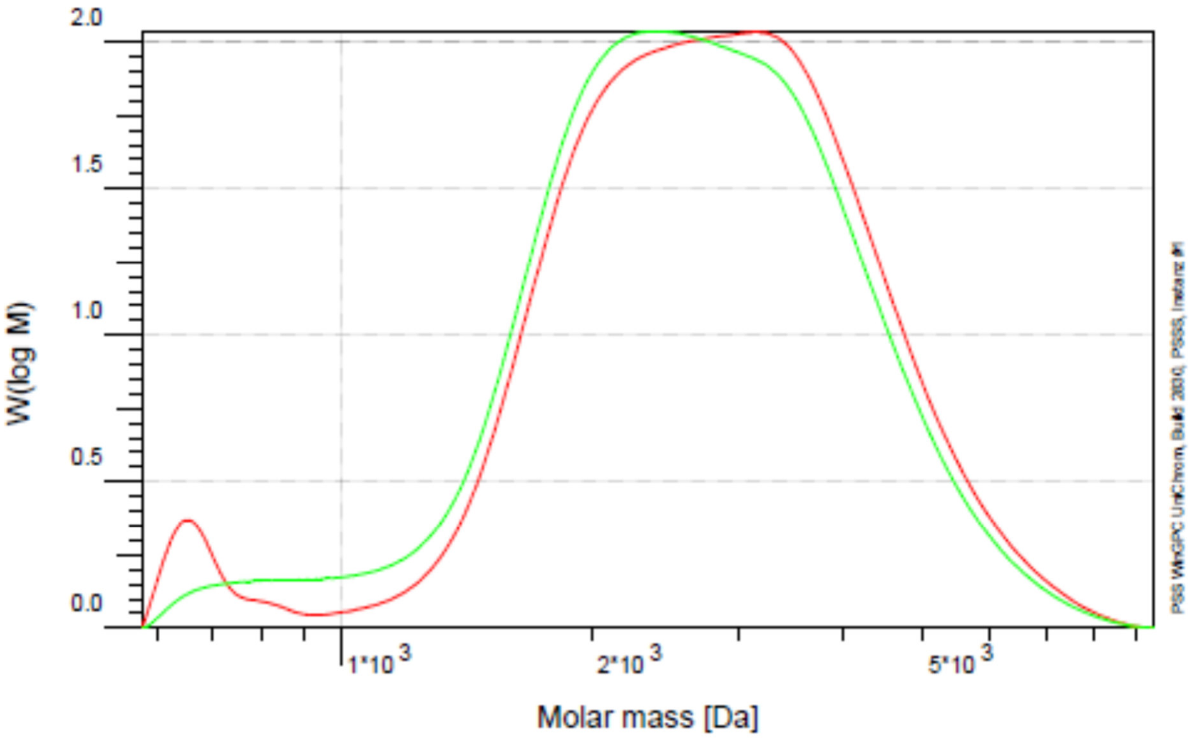

Gel permeation chromatography (GPC) trace of **15** (trial 1) (in THF); red line: detection by RI signal; green line: detection by UV signal.

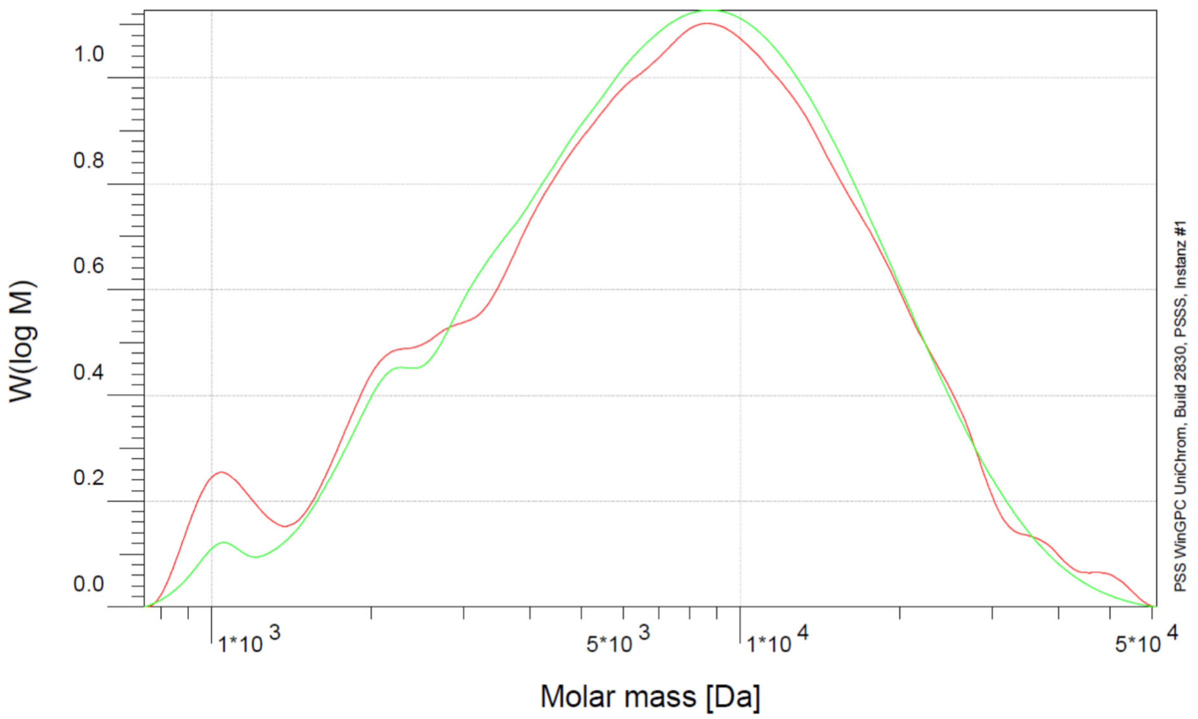

Gel permeation chromatography (GPC) trace of **15** (trial 2) (in THF); red line: detection by RI signal; green line: detection by UV signal.
